# Global, regional, and national trends in colorectal cancer burden from 1990 to 2021 and projections to 2040

**DOI:** 10.3389/fonc.2024.1466159

**Published:** 2025-01-16

**Authors:** Tao Zhang, Yuchen Guo, Binxu Qiu, Xianyu Dai, Yifei Wang, Xueyuan Cao

**Affiliations:** ^1^ Department of Gastric and Colorectal Surgery, General Surgery Center, The First Hospital of Jilin University, Changchun, Jilin, China; ^2^ Department of Urology, The First Hospital of Jilin University, Changchun, Jilin, China

**Keywords:** colorectal cancer, Global Burden of Disease, mortality, incidence, disability-adjusted life years

## Abstract

**Background:**

Colorectal cancer (CRC) is a common malignancy with notable recent shifts in its burden distribution. Current data on CRC burden can guide screening, early detection, and treatment strategies for efficient resource allocation.

**Methods:**

This study utilized data from the latest Global Burden of Diseases, Injuries, and Risk Factors (GBD) Study. Initially, a series of descriptive statistics were performed on the incident cases, deaths, disability-adjusted life years (DALYs), and age-standardized rates (ASRs) of CRC. Percentage changes and average annual percentage changes (AAPC) were then calculated to understand the trends in CRC disease burden. Decomposition and frontier analyses were conducted, and finally, the Bayesian age-period-cohort (BAPC) model was used to predict changes in ASRs up to 2040.

**Results:**

The GBD 2021 estimates indicate a significant increase in the global incident cases, deaths, and DALYs of CRC from 1990 to 2021. The age-standardized incidence rate (ASIR) increased (AAPC: 0.2), while the age-standardized mortality rate (ASMR) (AAPC: -0.72) and age-standardized DALYs rate (AAPC: -0.73) decreased. Males bore a higher disease burden than females, though the trends in disease burden changes were similar for both sexes. Although developed regions had higher incident cases, deaths, and DALYs, they showed more significant declines in ASRs. Decomposition analysis revealed that population growth and aging were the primary drivers of the increased disease burden. Frontier analysis showed that as the Socio-demographic Index increased, the disparity in CRC ASRs among countries widened, with developed regions having greater potential to reduce these rates. The By 2040, the BAPC model projects significant declines in global ASMR and age-standardized DALYs rates, while ASIR is expected to decrease in females but increase in males and across both sexes.

**Conclusion:**

CRC remains a significant public health issue with regional and gender differences, necessitating region- and population-specific prevention strategies.

## 1 Introduction

Colorectal cancer (CRC) is a common and deadly malignancy of the digestive system, posing a significant threat to global public health (1). According to data from the Global Cancer Observatory 2022, CRC is the third most common type of cancer worldwide, with over 1.9 million new cases and more than 900,000 deaths annually, making it the second leading cause of cancer-related deaths globally (2). The pathogenesis of CRC involves genetic mutations, environmental factors, and changes in the gut microbiome (3–5). Symptoms of CRC include rectal bleeding and changes in bowel habits, but these can also occur in healthy individuals, leading to low sensitivity for CRC detection (6). As a result, many patients are diagnosed at a late stage (7). Early detection and treatment can significantly improve survival rate (8).

The main risk factors for CRC include dietary habits, lifestyle, and genetic factors. High-fat diet, lack of physical activity, obesity, smoking, and alcohol consumption are known contributing factors (9, 10). Additionally, a family history and certain genetic syndromes, such as familial adenomatous polyposis and Lynch syndrome, increase the risk of developing CRC (11–13). The United Nations Sustainable Development Goal 3.4 aims to reduce premature mortality from non-communicable diseases, including cancer, by one-third by 2030 (14). Comprehensive assessment of CRC epidemiological trends can help implement targeted measures and track burden changes at global, regional, and national levels, providing guidance for future efforts.

Differences in CRC burden across countries and regions highlight the diversity and complexity of global response strategies. For example, in highly developed countries and regions, despite their advanced baseline levels, the incidence of CRC tends to stabilize or decline (15). However, in less developed countries and regions, the incidence is rapidly increasing due to rising risk factors (15). Governments and health organizations have taken steps to reduce the burden, such as promoting screening programs, encouraging healthy diets and lifestyles, and increasing public awareness of early symptoms (16, 17). While these measures have shown significant effects in some areas, screening and early diagnosis remain challenging in developing countries.

The Global Burden of Diseases, Injuries, and Risk Factors (GBD) Study is a comprehensive health data platform (18). The latest data, released in May 2024, covers 2021 and includes 288 causes of death, 371 diseases and injuries, and 88 risk factors across 204 countries and regions, providing estimates of incidence, mortality, disability-adjusted life years (DALYs) and other related metrics. This study aims to evaluate global CRC burden trends based on GBD 2021, integrating incidence, mortality, and DALYs indicators to clearly describe the distribution and trends of CRC at global, regional, and national levels, and to predict future CRC trends. These analyses not only help understand the current disease burden but also provide data support for future public health strategies and research directions. By identifying high-risk populations and regions, more targeted interventions can be developed to effectively reduce CRC incidence and mortality.

## 2 Materials and methods

### 2.1 Data sources

This study utilized data from the latest GBD study to systematically analyze the incidence, mortality, and DALYs of CRC from 1990 to 2021, covering global, regional, and national levels (18–20). GBD 2021 provides estimates for 204 countries and territories, which are grouped into 21 regions and 7 super-regions based on geographical proximity, epidemiological similarity, and cause-of-death distribution. The data are categorized by sex (both, female and male) and age (25 age groups from birth to 95 years and older), encompassing 371 diseases and injuries. The reported metrics include incidence, mortality, prevalence, years lived with disability, years of life lost, and DALYs. To ensure data consistency and comparability, disease burden estimates in the GBD study are based on the International Classification of Diseases codes. Detailed information can be found in the original literature.

### 2.2 Statistical analysis

This study obtained data on the number of new CRC cases from the GBD 2021 for the global population (both sexes, male, and female), 21 GBD regions, five Socio-demographic Index (SDI) groups, and 204 countries and territories for the years 1990 and 2021.The percentage change in the number of new cases from 1990 to 2021 was calculated using the formula: (2021 new cases - 1990 new cases)/1990 × 100% (21). Subsequently, the same method was applied to calculate the percentage changes in CRC deaths and disability-adjusted life years (DALYs) from 1990 to 2021. The study then gathered age-standardized incidence rates (ASIR), age-standardized mortality rates (ASMR), and age-standardized DALYs rates for CRC from GBD 2021 for the same demographics. The standard error (SE) was calculated using the formula: SE = (upper – lower)/(1.96 × 2), where upper and lower represent the upper and lower limits of the uncertainty interval (UI) for age-standardized rates (ASRs) obtained from GBD 2021 (22). Joinpoint regression analysis was performed using the Joinpoint software (Desktop version), provided by the National Cancer Institute (https://surveillance.cancer.gov/joinpoint/) (23, 24). The analysis began with exploratory analysis to identify overall trends and potential inflection points. The Joinpoint regression model fits the data with multiple potential inflection points, with each point dividing the time series into distinct linear segments. The slope of each segment represents the annual percentage change (APC) for that period. After selecting the optimal model, the trends and positions of each inflection point were described, and the APC for each segment was calculated, providing an annual average percentage change across multiple APCs. In our analysis, we employed the Permutation Test model. The APC, AAPC, and their confidence intervals (CIs) were calculated using the Parametric Method, while all other parameters were set to the default values provided by the desktop version of the Joinpoint software.

The Das Gupta method of decomposition analysis is an epidemiological tool for identifying the drivers of disease burden changes over time (25, 26). It decomposes changes in incident cases, mortality, and DALYs into three main components: epidemiological changes, population growth, and population aging. Epidemiological changes refer to shifts in disease incidence or mortality rates, reflecting medical advancements and public health improvements. Population growth refers to changes in the total population affecting disease burden, where rapid population growth can increase disease burden even if incidence and mortality rates remain constant. Population aging refers to the phenomenon where an increasing proportion of elderly individuals in the population may lead to a higher burden of chronic and non-communicable diseases.

Frontier analysis assesses the performance and efficiency of decision-making units (such as countries or regions) in converting inputs into outputs (27). This study employs Data Envelopment Analysis and Stochastic Frontier Analysis to construct an efficiency frontier representing best practices, comparing the performance of each decision-making unit against this frontier to determine efficiency levels. To analyze performance across different Socio-demographic Index (SDI) levels, we grouped the 204 countries and territories in the GBD study into five SDI categories: low, low-middle, middle, high-middle, and high SDI. We evaluated the performance of countries and territories within these groups, with the lower bound of ASIR, ASMR, and age-standardized DALYs rate representing the minimum values each country or territory could achieve based on their SDI level.

The Bayesian Age-Period-Cohort (BAPC) model, a Bayesian statistical method, analyzes and predicts demographic data by modeling the separate effects of age, period, and birth cohort (28). In the BAPC model, the age effect reflects the changing risk of events (like disease occurrence) as individuals age. The period effect considers environmental, policy, or other external influences affecting all individuals at a specific time point. The cohort effect refers to characteristics unique to people born in a particular period due to their distinct experiences and exposures. We used the BAPC R package to fit the model, predicting global ASR trends by gender up to 2040 (29).

Detailed procedural steps for the above analysis methods can be found in the supplementary methods section. Joinpoint regression analysis was performed using Joinpoint software (5.2.0.0), while other analyses were conducted in R (4.3.1). The data for this study were obtained from the Global Health Data Exchange GBD 2021 results website (https://vizhub.healthdata.org/gbd-results/), which are publicly available for free download and ethically approved in the original studies. Therefore, additional ethical approval was not required for our study. This study adheres to the strengthening the reporting of cohort, cross-sectional and case–control studies in surgery criteria (**Supplementary Table 1**).

## 3 Results

### 3.1 The burden and trend changes of CRC from 1990 to 2021

**Figure 1:**
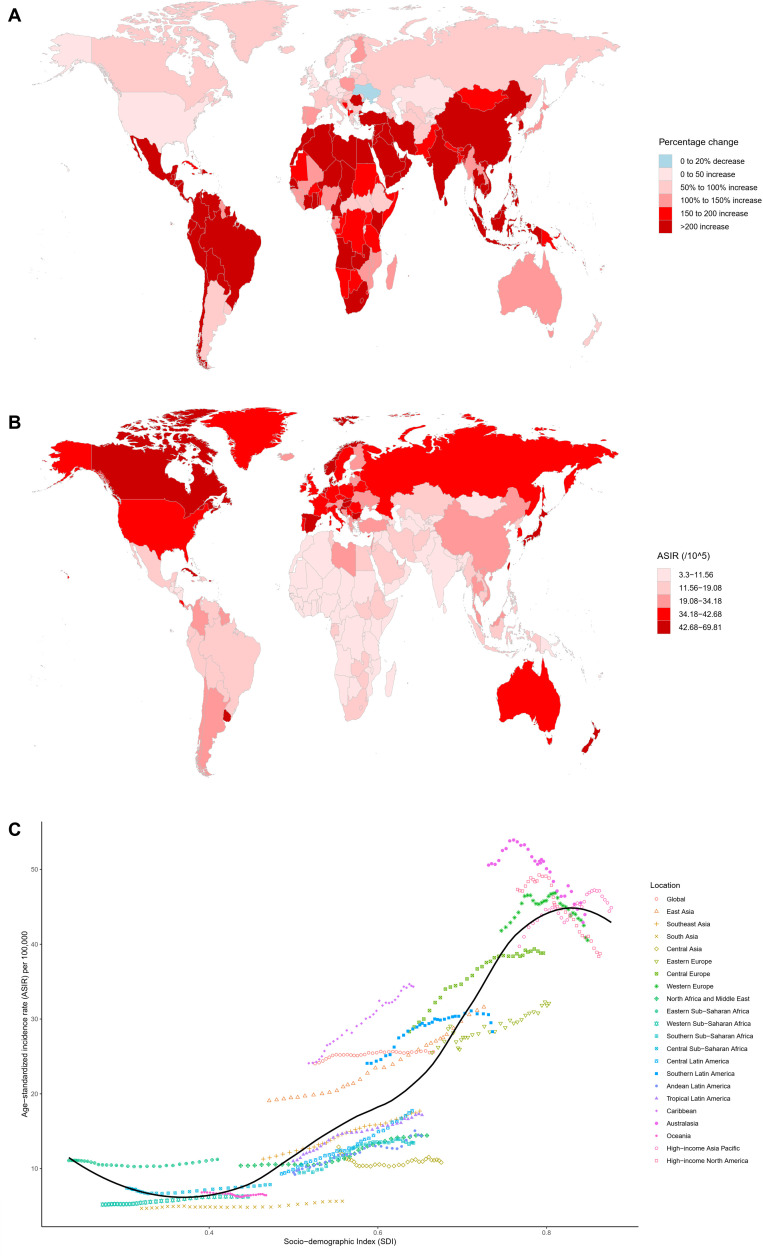
Multidimensional analysis of CRC incidence. **(A)** Percentage change in the number of incident cases from 1990 to 2021 across 204 countries and territories; **(B)** ASIR in 204 countries and territories in 2021; **(C)** Association between the SDI and ASIR in 21 GBD regions from 1990 to 2021. ASIR, age-standardized incidence rate; SDI, Socio-demographic index; CRC, colorectal cancer; GBD, Global Burden of Diseases, Injuries, and Risk Factors.

In 2021, a total of 2,194,143 new cases of CRC were reported globally, marking a 139.38% increase compared to 1990 (**Table 1**). The ASIR was 25.61 per 100,000 people, with an AAPC of 0.2 (95% CI: 0.16–0.25) (**Table 1**). Among the 21 GBD regions, East Asia recorded the highest number of new cases (684,927), accounting for 31.22% of the global total, followed by Western Europe, high-income North America, and high-income Asia Pacific (**Table 1**). Since 1990, all 21 GBD regions have shown an upward trend in incident cases, with increases ranging from 43.9% to 483.72% (**Table 1**). High-income Asia Pacific (44.89 per 100,000), Australasia (43.97 per 100,000), and Western Europe (40.54 per 100,000) reported the highest ASIR (**Table 1**). The AAPC among these regions varied significantly, ranging from -0.68 to 2.16 (**Table 1**). At the national level, China (mainland), the United States, and Japan had the highest numbers of new cases, collectively contributing 47.56% of the global total (**Supplementary Table 2**). Among 204 countries and territories, only Ukraine showed a decline in new case numbers, while others experienced varying degrees of increase, ranging from 3.64% to 1035.12% (**Figure 1A** and **Supplementary Table 2**). The Netherlands, Monaco, and Bermuda reported the highest ASIR globally, with AAPC ranging from -1.85 to 3.45 (**Figure 1B**, **Supplementary Figure 1A** and **Supplementary Table 2**). Notably, only 40 countries had an AAPC below zero (**Supplementary Table 2** and **Supplementary Figure 1A**). Gender differences revealed that males had 1.36 times the number of new cases as females, with an ASIR 1.58 times higher, and the burden of CRC improved more significantly for females (**Table 1**). From the perspective of the SDI, the ASIR in 204 countries and territories showed a significant increase with higher SDI levels (**Supplementary Figure 1B**). Between 1990 and 2021, ASIR trends across the 21 GBD regions exhibited fluctuations, first decreasing, then rising, and subsequently decreasing again (**Figure 1C**).

**Table 1:** Incident cases and ASIR of CRC in 1990 and 2021 by gender and GBD region.

Characteristics	Incident cases			ASIR per 100,000		
	1990 (95% UI)	2021 (95% UI)	Percentage change (%)	1990 (95% UI)	2021 (95% UI)	AAPC (95% CI)
Global	916583.53 (866238.31-951894.97)	2194143.25 (2001271.82-2359390.09)	139.38	24.04 (22.54-25.01)	25.61 (23.32-27.52)	0.2 (0.16-0.25)*
Sex						
Female	446820.89 (414136.02-472552.62)	930680.86 (824673.72-1017652.43)	108.29	21.41 (19.75-22.62)	20.17 (17.86-22.05)	-0.19 (-0.31 to -0.08)*
Male	469762.64 (445308.28-492302.5)	1263462.39 (1146499.47-1400377.23)	168.96	27.31 (25.89-28.51)	31.93 (29.04-35.26)	0.52 (0.47-0.56)*
21 GBD Regions						
East Asia	165083.41 (142141.86-189658.31)	684927.15 (559522.76-823301.33)	314.9	19.08 (16.55-21.79)	31.6 (25.9-37.85)	1.67 (1.51-1.83)*
Southeast Asia	29320.9 (24899.61-33179.32)	116941.67 (101259.79-132256.46)	298.83	11.28 (9.64-12.72)	17.7 (15.41-19.89)	1.48 (1.31-1.66)*
South Asia	28138.21 (24015.65-31933.64)	85115.13 (76613.73-95247.8)	202.49	4.69 (4-5.34)	5.65 (5.08-6.3)	0.63 (0.44-0.82)*
Central Asia	6180.16 (5774.01-6592.19)	8892.97 (7941.56-9809.25)	43.9	12.9 (12.03-13.79)	10.82 (9.69-11.9)	-0.47 (-0.73 to -0.2)*
Eastern Europe	71750.52 (69017.26-73942.57)	113252.18 (104414.53-122488.52)	57.84	25.52 (24.5-26.32)	32.11 (29.59-34.73)	0.77 (0.39-1.15)*
Central Europe	42181.94 (40229.86-43855.9)	85866.58 (79290.33-92786.95)	103.56	28.32 (27.03-29.45)	38.82 (35.71-41.96)	1.06 (0.81-1.31)*
Western Europe	243931.4 (229598.29-253377.82)	375461.84 (337713.28-401850.24)	53.92	41.81 (39.51-43.4)	40.54 (37.17-43.07)	-0.1 (-0.27-0.08)
North Africa and Middle East	17568.09 (14962.62-19789.76)	66086.55 (58129.63-74936.33)	276.17	10.4 (9.08-11.63)	14.43 (12.67-16.35)	1.08 (0.98-1.19)*
Eastern Sub-Saharan Africa	8179.36 (6322.72-9307.75)	17951.9 (15710.43-20779.07)	119.48	11.08 (8.75-12.51)	11.23 (9.84-12.77)	0.04 (-0.07-0.16)
Western Sub-Saharan Africa	4346.67 (3709.09-5101.1)	11620.39 (9673.14-13673.4)	167.34	5.2 (4.44-6.04)	6.29 (5.36-7.3)	0.62 (0.57-0.68)*
Southern Sub-Saharan Africa	2516.31 (2233.99-3056.7)	7623.41 (6875.23-8472.18)	202.96	9.46 (8.37-11.65)	13.45 (12.16-14.83)	1.08 (0.5-1.67)*
Central Sub-Saharan Africa	1590.92 (1268.4-1963.74)	4206.45 (3206.03-5573.58)	164.4	7.4 (6.06-9)	7.87 (6.09-10.53)	0.21 (0.14-0.27)*
Central Latin America	7632.33 (7310.61-7895.73)	44551.54 (39665.47-49720.64)	483.72	9.32 (8.9-9.67)	17.74 (15.75-19.81)	2.16 (2.04-2.28)*
Southern Latin America	10982.5 (10129.59-11892.01)	24746.82 (21921.77-27561.45)	125.33	24.09 (22.12-26.13)	28.32 (25.1-31.53)	0.54 (0.3-0.79)*
Andean Latin America	1902.38 (1647.69-2186.12)	8452.06 (6698.58-10526.05)	344.29	9.49 (8.24-10.87)	14.39 (11.39-17.89)	1.37 (0.63-2.1)*
Tropical Latin America	9843.2 (9351.15-10323.58)	44244.63 (40859.43-47143.63)	349.49	11 (10.32-11.56)	17.17 (15.82-18.31)	1.48 (1.31-1.66)*
Caribbean	6206.59 (5824.85-6606.77)	18481.22 (16074.15-20935.8)	197.77	24.09 (22.57-25.67)	34.33 (29.87-38.86)	1.2 (0.9-1.51)*
Australasia	11843.25 (10910.28-12803.79)	23280.74 (20501.57-26412.69)	96.57	50.58 (46.64-54.65)	43.97 (38.91-49.55)	-0.48 (-0.97-0.01)
Oceania	196.96 (161.74-238.77)	481.6 (409.98-560.14)	144.51	6.82 (5.75-8.03)	6.44 (5.55-7.42)	-0.19 (-0.32 to -0.06)*
High-income Asia Pacific	79543.27 (75502.03-82394.44)	207276.97 (179498.14-223331.93)	160.58	39.72 (37.43-41.17)	44.89 (40.2-47.85)	0.37 (0.13-0.62)*
High-income North America	167645.17 (155818.4-174737.06)	244681.45 (226550.01-256376.91)	45.95	47.34 (44.19-49.26)	38.75 (36.13-40.48)	-0.68 (-0.76 to -0.61)*
5 SDI Regions						
High SDI	475234.48 (449622.53-489570.48)	839754.51 (764451.14-885156.39)	76.7	42.79 (40.55-44.07)	40.52 (37.45-42.45)	-0.18 (-0.3 to -0.07)*
High-middle SDI	250999.09 (237455.6-263213.27)	669659.58 (598305.51-746397.46)	166.8	25.58 (24.12-26.81)	34 (30.33-37.95)	0.95 (0.82-1.08)*
Middle SDI	134432.24 (121314.77-148566.18)	526190.4 (462015.91-595123.93)	291.42	12.9 (11.65-14.16)	19.55 (17.14-22.04)	1.38 (1.25-1.51)*
Low-middle SDI	38285.8 (33160.41-43283.96)	119416.29 (109344.12-130918.56)	211.91	6.15 (5.36-6.94)	8.19 (7.51-8.96)	0.93 (0.87-0.99)*
Low SDI	16471.77 (12928.02-18870.81)	36649.31 (32707.36-40883.46)	122.5	7.32 (5.82-8.36)	7.39 (6.65-8.19)	0.05 (-0.03-0.13)

Statistically significant AAPC results are marked with an asterisk (*). ASIR, age-standardized incidence rate; CRC, colorectal cancer; GBD, Global Burden of Diseases, Injuries, and Risk Factors; UI, uncertainty interval; AAPC, average annual percent change; CI, confidence interval; SDI, Socio-demographic index.

In 2021, the global number of deaths caused by CRC reached 1,044,072, marking an 83.07% increase compared to 1990 (**Table 2**). The global ASMR was 12.40 per 100,000 population, with a declining trend in CRC ASMR since 1990 (AAPC: -0.72, 95% CI: -0.81 to -0.63) (**Table 2**). Among the 21 GBD regions, East Asia reported the highest number of deaths at 287,880, followed by Western Europe, high-income North America, and high-income Asia Pacific (**Table 2**). Compared to 1990, all GBD regions saw an increase in deaths, ranging from 14.86% to 308.39% (**Table 2**). Central Europe (22.58 per 100,000), Southern Latin America (18.13 per 100,000), and Eastern Europe (18.05 per 100,000) recorded the highest ASMRs, with about half of the regions showing declining ASMR trends, with AAPC variations ranging from -1.76 to 0.85 (**Table 2**). At the national level, China (mainland), the United States, and Japan had the highest death counts, with China accounting for 26.35% of global deaths (**Table 2**). Compared to 1990, the percentage change in deaths by country ranged significantly, spanning from -22.4% to 609.49% (**Supplementary Table 3** and **Supplementary Figure 2A**). Uruguay, Hungary, and Bulgaria reported the highest ASMRs (**Supplementary Table 3** and **Supplementary Figure 2B**), while nearly half of the countries experienced a decline in ASMR, with AAPC values ranging from -2.78 to 3.07 (**Supplementary Table 3** and **Supplementary Figure 3A**). Gender-wise, male deaths numbered 581,557, 1.26 times higher than females at 462,515, with male ASMR at 15.35 per 100,000, 1.54 times higher than females (9.96 per 100,000) (**Table 2**). Male CRC deaths and ASMR improvements were less pronounced compared to females (**Table 2**). At the SDI level, the ASMR of 204 countries and territories in 2021 showed an initial increase followed by a decrease with rising SDI levels (**Supplementary Figure 3B**), while from 1990 to 2021, ASMR trends across the 21 GBD regions experienced a decline, followed by an increase, and then a subsequent decline with increasing SDI (**Supplementary Figure 2C**).

**Table 2:** Death cases and ASMR of CRC in 1990 and 2021 by gender and GBD region.

Characteristics	Death cases			ASMR per 100,000		
	1990 (95% UI)	2021 (95% UI)	Percentage change (%)	1990 (95% UI)	2021 (95% UI)	AAPC (95% CI)
Global	570318.55 (536544.84-597668.66)	1044072.21 (950187.61-1120169.34)	83.07	15.56 (14.49-16.31)	12.4 (11.24-13.31)	-0.72 (-0.81 to -0.63)*
Sex						
Female	282607.87 (258885.98-301416.1)	462514.95 (407296.33-503539.24)	63.66	13.89 (12.68-14.8)	9.96 (8.78-10.84)	-1.07 (-1.17 to -0.97)*
Male	287710.67 (269819.1-304982.31)	581557.26 (528252.82-641420.11)	102.13	17.72 (16.67-18.68)	15.35 (13.94-16.87)	-0.45 (-0.54 to -0.37)*
21 GBD Regions						
East Asia	123638.04 (106930.29-141856.96)	287880.01 (235559.37-343280.49)	132.84	15.43 (13.39-17.61)	13.78 (11.33-16.35)	-0.37 (-0.52 to -0.21)*
Southeast Asia	24777.24 (20969.45-28065.26)	79419.6 (68449.69-89290.46)	220.53	10.11 (8.66-11.38)	12.74 (11.12-14.26)	0.75 (0.62-0.87)*
South Asia	25281.24 (21597.24-28740.57)	66942.71 (60196.67-74844.4)	164.79	4.42 (3.76-5.06)	4.63 (4.17-5.16)	0.17 (-0.1-0.44)
Central Asia	4831.31 (4498.76-5156.83)	6146.25 (5492.55-6771.9)	27.22	10.35 (9.61-11.06)	7.87 (7.06-8.65)	-0.74 (-1.22 to -0.25)*
Eastern Europe	50096.8 (48178.39-51687.69)	64373.25 (59115.5-69822.45)	28.5	18.06 (17.33-18.65)	18.05 (16.58-19.56)	-0.04 (-0.52-0.46)
Central Europe	32120.12 (30603.11-33395.98)	51843.49 (47752.16-55681.29)	61.41	22.04 (21-22.93)	22.58 (20.81-24.28)	0.08 (-0.11-0.28)
Western Europe	136376.65 (127107.86-142174.17)	156637.47 (136856.63-169938.67)	14.86	22.98 (21.45-23.93)	15.11 (13.53-16.25)	-1.35 (-1.49 to -1.22)*
North Africa and Middle East	14108.39 (12125.66-15839.38)	37394.51 (32758.79-42274.13)	165.05	8.99 (7.94-10.06)	8.95 (7.85-10.08)	0 (-0.14-0.14)
Eastern Sub-Saharan Africa	7778.77 (5997.52-8844.28)	15967.48 (13939.89-18320.33)	105.27	11.06 (8.71-12.49)	10.76 (9.41-12.15)	-0.09 (-0.21-0.04)
Western Sub-Saharan Africa	4161.81 (3556.9-4867.26)	10312.43 (8668.12-12099.87)	147.79	5.22 (4.48-6.04)	5.98 (5.12-6.88)	0.44 (0.36-0.52)*
Southern Sub-Saharan Africa	2225.26 (1974.24-2720.01)	6129.62 (5550.19-6785.91)	175.46	8.82 (7.79-10.94)	11.47 (10.37-12.61)	0.8 (0.23-1.37)*
Central Sub-Saharan Africa	1493.97 (1200.08-1837.78)	3686.77 (2802.24-4932)	146.78	7.43 (6.05-9.04)	7.45 (5.77-10.14)	0.02 (-0.04-0.08)
Central Latin America	5615.77 (5367.62-5819.03)	22934.27 (20341.66-25521.14)	308.39	7.21 (6.85-7.49)	9.3 (8.26-10.35)	0.85 (0.73-0.97)*
Southern Latin America	8973.71 (8273.58-9706.28)	16117.02 (14307.19-18001.8)	79.6	20.13 (18.48-21.81)	18.13 (16.12-20.25)	-0.32 (-0.54 to -0.09)*
Andean Latin America	1722.56 (1495.5-1967.13)	5777.84 (4597.21-7047.45)	235.42	8.88 (7.71-10.08)	10 (7.98-12.18)	0.36 (-0.47-1.2)
Tropical Latin America	8102.19 (7660.32-8506.31)	29414.56 (26980.72-31338.9)	263.04	9.55 (8.91-10.06)	11.58 (10.59-12.34)	0.69 (0.49-0.9)*
Caribbean	3471.99 (3238.58-3720.89)	7881.54 (6866.69-8970.24)	127	13.96 (13.01-14.95)	14.57 (12.72-16.6)	0.29 (0.25-0.33)*
Australasia	5777.75 (5304.53-6246.04)	8275.77 (7178.89-9408.21)	43.24	24.88 (22.77-26.91)	14.62 (12.77-16.52)	-1.76 (-2.14 to -1.38)*
Oceania	165.31 (136.17-200.22)	381.66 (324.23-445.96)	130.87	6.31 (5.37-7.42)	5.58 (4.8-6.42)	-0.41 (-0.51 to -0.31)*
High-income Asia Pacific	35635.73 (33642.83-36953.94)	80690.59 (67266.65-88001.37)	126.43	18.45 (17.23-19.2)	14.99 (13.03-16.09)	-0.68 (-0.91 to -0.45)*
High-income North America	73963.93 (67846.12-77459.92)	85865.38 (77871.23-90928.14)	16.09	20.58 (18.95-21.52)	12.95 (11.89-13.65)	-1.52 (-1.63 to -1.41)*
5 SDI Regions						
High SDI	243217.48 (227487.51-251652.23)	336566.14 (299021.83-359612.84)	38.38	21.86 (20.43-22.64)	15.02 (13.58-15.92)	-1.21 (-1.27 to -1.14)*
High-middle SDI	171222.14 (160927-179669.98)	308174.8 (278041.59-338271.33)	79.99	18.12 (17.02-19.01)	15.71 (14.14-17.25)	-0.44 (-0.62 to -0.27)*
Middle SDI	105724.63 (95513.39-116510.55)	274871.22 (243369.11-306694.04)	159.99	10.79 (9.76-11.82)	10.64 (9.42-11.84)	-0.05 (-0.18-0.09)
Low-middle SDI	33887.42 (29282.28-38355.52)	91274.17 (83732.61-99854.47)	169.35	5.72 (4.99-6.45)	6.55 (6.02-7.17)	0.45 (0.37-0.53)*
Low SDI	15461.63 (12105.52-17686.55)	31847.59 (28503.9-35514.27)	105.98	7.21 (5.75-8.21)	6.88 (6.18-7.62)	-0.13 (-0.24 to -0.02)*

Statistically significant AAPC results are marked with an asterisk (*). ASMR, age-standardized mortality rate; CRC, colorectal cancer; GBD, Global Burden of Diseases, Injuries, and Risk Factors; UI, uncertainty interval; AAPC, average annual percent change; CI, confidence interval; SDI, Socio-demographic index.

In 2021, CRC globally caused a total of 24,401,100 DALYs, representing a 69.49% increase compared to 1990 (**Table 3**). The global age-standardized DALYs rate was 283.24 per 100,000 population, showing a decreasing trend compared to 1990 (AAPC: -0.73, 95% CI: -0.82 to -0.64) (**Table 3**). Among the 21 GBD regions, East Asia, Western Europe, and Southeast Asia had the highest DALY numbers, with East Asia accounting for 29.3% of the global total (**Table 3**). Compared to 1990, all 21 regions experienced an increase in DALY numbers, with growth ranging from 3.44% to 298% (**Table 3**). Central Europe had the highest age-standardized DALY rate in 2021 at 506.48 per 100,000 population, followed by Eastern Europe, Southern Latin America, and the Caribbean (**Table 3**). The AAPC of age-standardized DALYs rate across these regions ranged from -1.83 to 1.14 (**Table 3**). At the national level, China, the United States, and India had the highest DALY numbers in 2021, with changes from 1990 ranging from -28.57% to 581.51%; only nine countries showed a decline in DALYs (**Supplementary Table 4** and **Supplementary Figure 4A**). Hungary, Bulgaria, and Uruguay recorded the highest age-standardized DALYs rate globally (**Supplementary Table 4** and **Supplementary Figure 4B**), with AAPC changes across 204 countries and regions ranging from -2.53 to 2.71(**Supplementary Table 4** and **Supplementary Figure 5A**). The age-standardized DALYs rate for males was 349.67 per 100,000 population, significantly higher than the 224.3 per 100,000 for females, with females showing greater improvement in both DALYs and age-standardized DALY rates (**Table 3**). At the level of the SDI, in 2021, the age-standardized DALYs rate across 204 countries showed a trend of first increasing and then decreasing with rising SDI (**Supplementary Figure 5B**). From 1990 to 2021, the age-standardized DALYs rate across the 21 GBD regions demonstrated a trend of first declining, then rising, and subsequently declining again with changes in SDI (**Supplementary Figure 4C**).

**Table 3:** DALYs and age-standardized DALYs rate of CRC in 1990 and 2021 by gender and GBD region.

Characteristics	DALYs			Age-standardized DALYs rate per 100,000		
	1990 (95% UI)	2021 (95% UI)	Percentage change (%)	1990 (95% UI)	2021 (95% UI)	AAPC (95% CI)
Global	14396657.72 (13568749.36-15166575.84)	24401100.18 (22689368.55-26161517.73)	69.49	357.33 (336.62-375.74)	283.24 (263.11-303.33)	-0.73 (-0.82 to -0.64)*
Sex						
Female	6786954.89 (6292858.34-7304106.47)	10233850.84 (9257555.91-11064615.26)	50.79	316.54 (292.93-340.38)	224.3 (203.21-242.65)	-1.1 (-1.18 to -1.02)*
Male	7609702.83 (7037903.25-8139914.51)	14167249.34 (12782331.8-15683970.51)	86.17	405.58 (378.49-431.93)	349.67 (316.68-386.64)	-0.46 (-0.56 to -0.36)*
21 GBD Regions						
East Asia	3691551.51 (3152648.28-4246182.29)	7148995.32 (5822935.26-8561078.94)	93.66	389.57 (334.25-446.9)	334.5 (274.01-399.93)	-0.49 (-0.6 to -0.37)*
Southeast Asia	735821.83 (612421.7-841711.89)	2166650.37 (1868880.14-2456349.67)	194.45	257.93 (218.13-292.17)	313.37 (270.75-353.54)	0.63 (0.59-0.66)*
South Asia	787250.63 (675897.9-889379.65)	1908668.14 (1711982.01-2155055.24)	142.45	119.12 (101.91-135.16)	120.37 (108.3-135.3)	0.06 (-0.05-0.16)
Central Asia	140373.97 (132360.71-149146.15)	169952.25 (151537.43-187708)	21.07	280.69 (263.92-298.74)	196.55 (175.59-216.68)	-0.99 (-1.11 to -0.86)*
Eastern Europe	1280866.45 (1237775.47-1320907.1)	1465104.15 (1343980.89-1600585.46)	14.38	455.74 (440.17-469.53)	424.54 (389.69-463.67)	-0.28 (-0.85-0.3)
Central Europe	767091.48 (735174.35-795936.07)	1087562.43 (1003626.42-1168378.1)	41.78	512.39 (489.92-531.48)	506.48 (467.97-544.57)	-0.02 (-0.19-0.14)
Western Europe	2815471.87 (2680130.07-2917394.76)	2912355.2 (2641021.36-3112518.77)	3.44	498.57 (476.35-516.24)	326.82 (301.67-347.23)	-1.38 (-1.54 to -1.22)*
North Africa and Middle East	409788.25 (341302.19-464924)	1012651.6 (886198.88-1154502.81)	147.12	220.98 (187.75-248.96)	209.04 (183.16-237.28)	-0.18 (-0.29 to -0.07)*
Eastern Sub-Saharan Africa	225496.31 (171119.13-258758.96)	444252.95 (385799.78-525458.32)	97.01	274.48 (211.05-312.21)	242.13 (211.3-279.08)	-0.43 (-0.53 to -0.32)*
Western Sub-Saharan Africa	110380.88 (93741.12-130867.07)	276692.44 (224567.81-328426.26)	150.67	120.11 (102.58-141.26)	132.05 (110.39-155.48)	0.31 (0.25-0.36)*
Southern Sub-Saharan Africa	60914.31 (54661.61-72328.1)	166061.78 (149959.4-186753.43)	172.62	207.84 (185.42-251.15)	270.64 (244.94-301.85)	0.78 (0.1-1.47)*
Central Sub-Saharan Africa	44003.07 (35102.63-54650.24)	109622.05 (82688.06-146971.08)	149.12	179.38 (145.64-220.18)	177.79 (135.17-238.59)	-0.02 (-0.09-0.05)
Central Latin America	149274.84 (143940.78-154014.73)	594116.79 (529274.15-662367.07)	298	165.76 (159.24-171.33)	231.95 (206.64-258.57)	1.14 (1.05-1.22)*
Southern Latin America	206237.38 (189818.49-224030.55)	349581.13 (311369.42-391893.39)	69.5	445.86 (410.18-484.75)	407.99 (363.05-457.56)	-0.27 (-0.55-0.01)
Andean Latin America	43988.07 (37837.61-50560.74)	136183.21 (108534.69-167981.61)	209.59	203.47 (175.15-233.97)	226.76 (180.59-279.8)	0.33 (-0.45-1.12)
Tropical Latin America	218356.84 (209208.99-228658.47)	744957.01 (694956.43-786849.09)	241.16	224.28 (213.27-235.19)	286.22 (266.67-302.45)	0.82 (0.68-0.96)*
Caribbean	83679.93 (78049.17-89688.93)	179751.92 (155874.18-205420.07)	114.81	317.26 (295.9-339.95)	335.02 (290.41-383.11)	0.26 (0.1-0.42)*
Australasia	133623.41 (123838.12-143847.28)	166740.1 (147523.69-186890.83)	24.78	577.93 (535.91-621.91)	327.19 (290.9-364.68)	-1.83 (-2 to -1.66)*
Oceania	5152.78 (4141.84-6362.22)	11642.97 (9764-13750.35)	125.96	154.39 (127.38-186.63)	136.65 (116.1-159.45)	-0.42 (-0.57 to -0.26)*
High-income Asia Pacific	874132.26 (837040.69-905134.7)	1441630 (1272495.98-1549758.69)	64.92	431.84 (411.77-447.55)	331.48 (303.85-352.47)	-0.86 (-1.05 to -0.68)*
High-income North America	1613201.65 (1529313.04-1679101.05)	1907928.37 (1788110.92-2003823.63)	18.27	469.38 (446.3-487.53)	316.04 (298.52-330.84)	-1.28 (-1.59 to -0.98)*
5 SDI Regions						
High SDI	5344610.88 (5120390.32-5504892.2)	6705748.75 (6179255.7-7070831.33)	25.47	490.5 (470.41-505.01)	338.23 (316.75-354.91)	-1.21 (-1.33 to -1.09)*
High-middle SDI	4416013.34 (4153232.36-4663023.04)	7079677.99 (6397925.01-7847047.95)	60.32	436.82 (410.01-460.37)	364.61 (329.26-404.77)	-0.59 (-0.78 to -0.39)*
Middle SDI	3142817.93 (2820037.33-3490093.55)	7120502.07 (6308564.18-7920507.32)	126.56	273.63 (246.6-302.13)	259.35 (230.17-288.24)	-0.18 (-0.29 to -0.06)*
Low-middle SDI	1021298.78 (879103.56-1160913.8)	2564120.92 (2333600.66-2815224)	151.06	149.42 (129.02-169.5)	166.47 (151.97-182.5)	0.36 (0.31-0.41)*
Low SDI	452673.17 (350578.1-521203.2)	901644.93 (799642.29-1014005.07)	99.18	181.87 (142.04-208.32)	162.29 (145.25-181.89)	-0.36 (-0.44 to -0.28)*

Statistically significant AAPC results are marked with an asterisk (*). DALYs, disability-adjusted life years; CRC, colorectal cancer; GBD, Global Burden of Diseases, Injuries, and Risk Factors; UI, uncertainty interval; AAPC, average annual percent change; CI, confidence interval; SDI, Socio-demographic Index.

### 3.2 Trends in the burden of CRC from 1990 to 2021 by continent and gender

**Figure 2:**
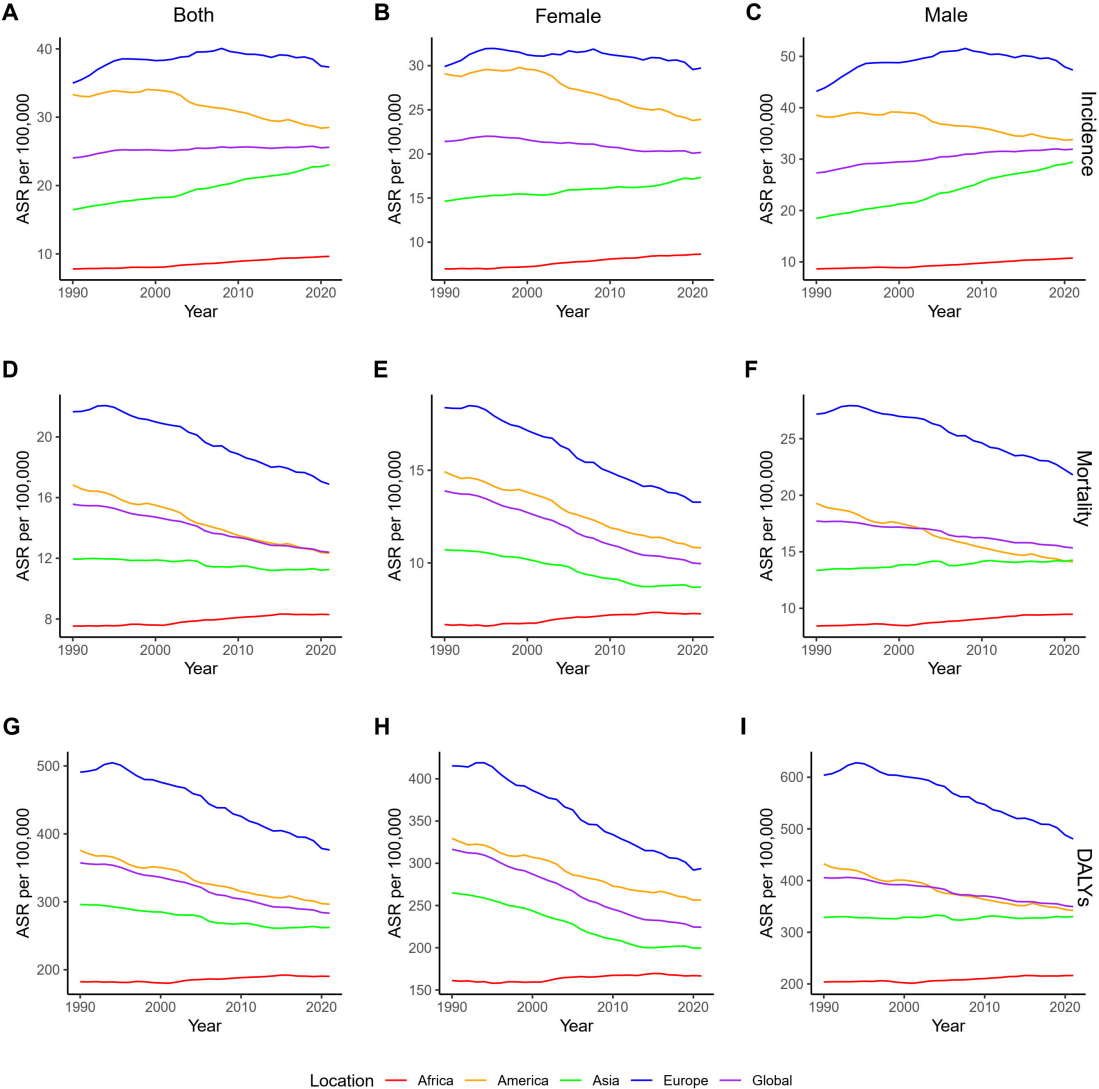
ASRs of CRC by continent and gender from 1990 to 2021. **(A–C)**. ASIRs for all sexes, females, and males; **(D–F)**. ASMRs for all sexes, females, and males; **(G–I)**. Age-standardized DALYs rate for all sexes, females, and males. DALYs, disability-adjusted life years; ASRs, age-standardized rates; CRC, colorectal cancer; ASIRs, age-standardized incidence rates; ASMR, age-standardized mortality rates.

The analysis of global CRC burden from 1990 to 2021 based on GBD 2021 data, divided by four continents, shows the following trends. In terms of ASIR, Europe and the Americas have higher ASIRs than the global average in both sexes, females and males, while Asia and Africa have lower ASIRs (**Figures 2A–C**). From a trend perspective, both Asia and Africa show an increasing ASIR for both sexes, females and males, whereas the Americas exhibit a decreasing trend (**Supplementary Figure 6A**). In Europe and globally, a statistically significant increase in ASIR was observed in both sexes and in males, while the global female ASIR significantly declined, with no significant changes observed in European females; additionally, a statistically significant decline was observed in global females (**Supplementary Figure 6A**). Regarding ASMR, Asia and Africa have lower ASMRs than the global average in both sexes and females, Europe has a higher ASMR, and the Americas show a more complex pattern: female ASMR has consistently been higher than the global average, while both sex ASMR has approached the global level since 2010, and male ASMR has been lower than the global level since 2003 (**Figures 2D–F**). Between 1990 and 2021, ASMR showed a downward trend for all sexes in the global, Americas, and European regions, while in Asia, the all-sex and female ASMR also decreased, but in Africa and in Asian males, ASMR increased (**Supplementary Figure 6B**). In terms of age-standardized DALYs rate, the Americas have consistently had a higher DALYs rate than the global average, while Asian males, who have never surpassed the Americas, showed no significant change (**Figures 2G–I** and **Supplementary Figure 6C**). The DALYs rate distribution and trends in other regions follow similar patterns to those of ASMR (**Figures 2G–I** and **Supplementary Figure 6C**).

### 3.3 Decomposition and Frontier analysis of CRC

**Figure 3:**
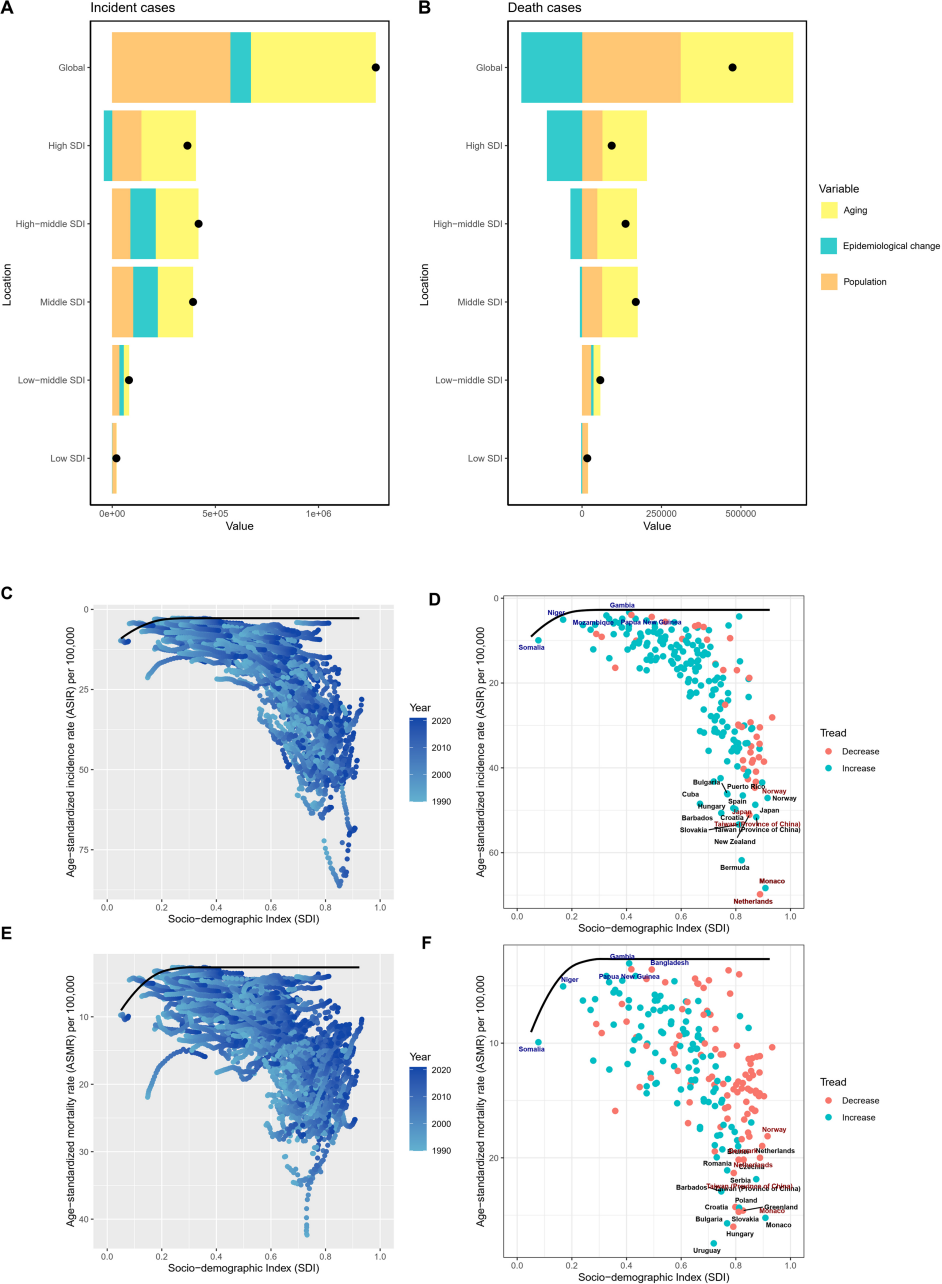
Decomposition and frontier analysis results of CRC incidence and mortality. **(A)** Decomposition analysis of incident cases from 1990 to 2021; **(B)** Decomposition analysis of death cases from 1990 to 2021. Black dots represent the overall changes in disease burden due to aging, epidemiological changes, and population growth. For each component, an increase in the disease burden of CRC related to that component is indicated by positive values, whereas a decrease is indicated by negative values. **(C, D)**. Frontier analysis of ASIR from 1990 to 2021; **(E, F)**. Frontier analysis of ASMR from 1990 to 2021. Black lines represent the lower limits of ASR achievable at different SDI levels, with points representing different countries and regions. The 15 countries and regions with the largest effective differences globally are labeled in black font, the 5 countries and regions with the smallest effective differences among low SDI countries are labeled in blue font, and the 5 countries and regions with the largest effective differences among high SDI countries are labeled in red font. In Figures **(C, E)**, the blue dots represent the ASRs of CRC from 1990 to 2021, with darker shades indicating later years. In Figures **(D, F)**, the dots represent changes in CRC ASR from 1990 to 2021. Blue dots indicate countries and territories where the ASR increased from 1990 to 2021, while red dots indicate countries and territories where the ASR decreased. SDI, Socio-demographic index; ASIR, age-standardized incidence rate; ASMR, age-standardized mortality rate; CRC, colorectal cancer; ASR, age-standardized rate.

Decomposition analysis indicated significant increases in global incident cases, death cases, and DALYs from 1990 to 2021, with population growth and aging playing a leading role in these trends (**Table 4**, **Figures 3A, B** and **Supplementary Figure 7**). The increase was notably higher in middle, high-middle, and high SDI regions compared to low and low-middle SDI regions (**Table 4**, **Figures 3A, B** and **Supplementary Figure 7**). In low, middle, high-middle, and high SDI regions, epidemiological trends negatively impacted ASMR and the age-standardized DALYs rate, while population growth and aging played a promoting role, unlike in the low-middle SDI region (**Table 4**, **Figure 3B**, and **Supplementary Figure 7**).

**Table 4:** Detailed results of the decomposition analysis for CRC.

Measure	Location	Overall difference	Change due to population-level determinants (% contribute to the total changes)		
			Aging	Population	Epidemiological change
Incident cases	Global	1277559.716	606039.19 (47.44)	572975.78 (44.85)	98544.75 (7.71)
	High SDI	364520.025	264391.37 (72.53)	140913.42 (38.66)	-40784.77 (-11.19)
	High-middle SDI	418660.495	208315.06 (49.76)	88127.58 (21.05)	122217.85 (29.19)
	Middle SDI	391758.1571	170936.52 (43.63)	102100.52 (26.06)	118721.12 (30.3)
	Low-middle SDI	81130.49646	25414.93 (31.33)	35541.94 (43.81)	20173.63 (24.87)
	Low SDI	20177.54273	794.49 (3.94)	20203.02 (100.13)	-819.96 (-4.06)
Death cases	Global	473753.6641	354101.01 (74.74)	310809.05 (65.61)	-191156.39 (-40.35)
	High SDI	93348.66594	139534.65 (149.48)	64277.44 (68.86)	-110463.42 (-118.33)
	High-middle SDI	136952.659	124780.97 (91.11)	48257.77 (35.24)	-36086.08 (-26.35)
	Middle SDI	169146.587	112383.66 (66.44)	62997.07 (37.24)	-6234.15 (-3.69)
	Low-middle SDI	57386.75418	21570.55 (37.59)	28891.45 (50.35)	6924.75 (12.07)
	Low SDI	16385.95818	813.49 (4.96)	18260.58 (111.44)	-2688.11 (-16.4)
DALYs	Global	10004442.46	7133487.38 (71.3)	7507130.2 (75.04)	-4636175.13 (-46.34)
	High SDI	1361137.875	2332014.89 (171.33)	1338932.03 (98.37)	-2309809.05 (-169.7)
	High-middle SDI	2663664.649	2552935.02 (95.84)	1164794.58 (43.73)	-1054064.95 (-39.57)
	Middle SDI	3977684.133	2605551.37 (65.5)	1720220.72 (43.25)	-348087.95 (-8.75)
	Low-middle SDI	1542822.134	546505.09 (35.42)	835978.97 (54.19)	160338.07 (10.39)
	Low SDI	448971.7663	21495.97 (4.79)	526698.55 (117.31)	-99222.75 (-22.1)

CRC, colorectal cancer; SDI, Socio-demographic Index; DALYs, disability-adjusted life years.

In **Figures 3C–F** and **Supplementary Figure 8**, the results of the frontier analysis are visualized. The black line represents the lowest achievable ASRs value for each country or region at its current SDI level. The distance of each point from the black line indicates the potential for future reduction in disease burden, with greater distances suggesting greater potential. Across the six plots, the points form an inverted triangle shape, with lower values on the left and higher values on the right. As SDI increases, the range of point distribution also expands, suggesting that countries with higher SDI levels may have greater potential for reducing SDI. The analysis identifies the 15 globally worst-performing countries, the 5 best-performing countries in low SDI regions, and the 5 worst-performing countries in high SDI regions in **Figures 3C-F** and **Supplementary Figure 8**. This study presents the detailed results of the analysis in **Supplementary Tables 5**–**7**.

### 3.4 Projections of ASRs of CRC to 2040

**Figure 4:**
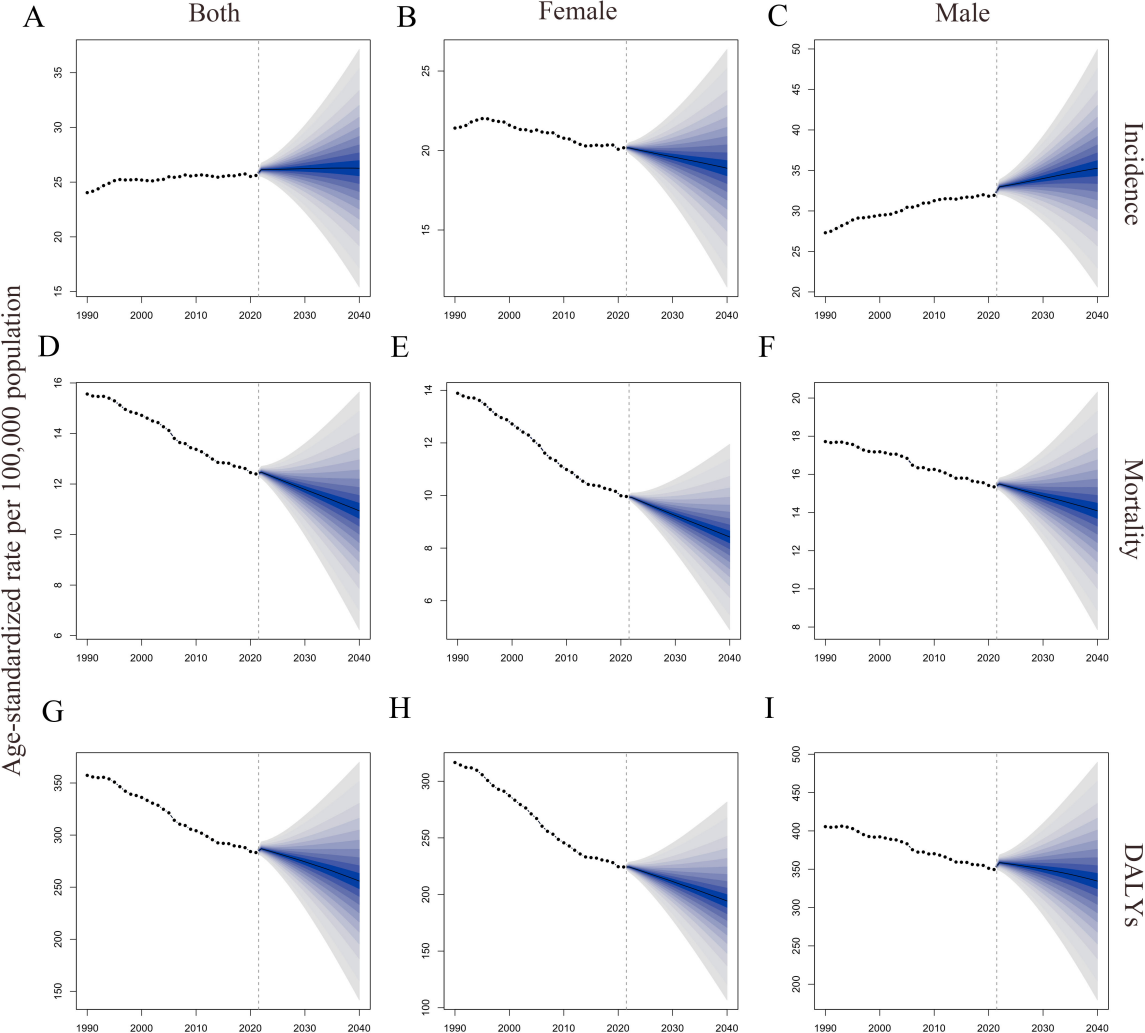
Global trends in ASRs of CRC by gender from 1990 to 2040. **(A–C)**. ASIRs for all sexes, females, and males; **(D–F)**. ASMRs for all sexes, females, and males; **(G–I)**. Age-standardized DALYs rate for all sexes, females, and males. DALYs, disability-adjusted life years; ASRs, age-standardized rates; CRC, colorectal cancer; ASIRs, age-standardized incidence rates; ASMRs, age-standardized mortality rates.

Using the BAPC model, we projected trends in ASIR, ASMR, and age-standardized DALYs rate for CRC from 2022 to 2040 by gender (**Table 5** and **Figure 4**). Predictions indicate that ASIR increases in both sexes and in males and decreases in females. Both ASMR and age-standardized DALYs rate were projected to decline across gender groups, showing similar trends.

**Table 5:** Prediction of ASRs from 2020 to 2040.

Year	ASIR			ASMR			Age-standardized DALYs rate		
	Both	Female	Male	Both	Female	Male	Both	Female	Male
2022	26.14	20.16	32.98	12.47	9.92	15.49	286.85	224.29	358.66
2023	26.14	20.09	33.1	12.38	9.84	15.42	285.39	222.74	357.74
2024	26.15	20.01	33.22	12.3	9.75	15.34	283.89	221.17	356.76
2025	26.17	19.94	33.34	12.21	9.66	15.26	282.34	219.54	355.72
2026	26.18	19.88	33.47	12.12	9.58	15.18	280.75	217.9	354.64
2027	26.19	19.8	33.61	12.04	9.49	15.1	279.17	216.28	353.59
2028	26.21	19.73	33.74	11.95	9.41	15.03	277.61	214.7	352.51
2029	26.22	19.67	33.87	11.87	9.32	14.95	276.01	213.1	351.37
2030	26.23	19.6	34	11.79	9.24	14.87	274.35	211.45	350.15
2031	26.24	19.53	34.14	11.7	9.16	14.8	272.62	209.76	348.85
2032	26.25	19.46	34.27	11.62	9.08	14.72	270.89	208.07	347.54
2033	26.26	19.39	34.41	11.54	8.99	14.65	269.18	206.43	346.21
2034	26.27	19.32	34.54	11.45	8.91	14.57	267.44	204.79	344.81
2035	26.28	19.26	34.67	11.37	8.83	14.49	265.62	203.08	343.3
2036	26.28	19.19	34.8	11.28	8.75	14.41	263.69	201.3	341.65
2037	26.28	19.11	34.92	11.2	8.67	14.34	261.75	199.52	339.96
2038	26.28	19.04	35.05	11.11	8.59	14.26	259.82	197.79	338.24
2039	26.28	18.96	35.17	11.03	8.51	14.18	257.88	196.07	336.46
2040	26.27	18.89	35.27	10.94	8.42	14.09	255.83	194.27	334.53

ASRs, age-standardized rates; ASIR, age-standardized incidence rate; ASMR, age-standardized mortality rate; DALYs, disability-adjusted life years.

## 4 Discussion

Our study systematically analyzed the global burden of CRC, revealing significant geographical and gender differences in incidence, mortality, and DALYs, and projecting future trends to 2040. In 2021, there were approximately 2,194,143 new cases of CRC, 1,044,072 deaths, and a total of 24,401,100 DALYs lost worldwide. From 1990 to 2021, while the global ASIR slightly increased, the ASMR and age-standardized DALYs rate significantly declined. Men bore a significantly higher burden of CRC than women. Considering the SDI, countries with higher SDI had higher incident cases, mortality, and DALYs, but also showed greater declines in ASMR and age-standardized DALYs rate, particularly in regions such as Europe and high-income North America. Conversely, less developed regions such as Africa, South Asia, and Southeast Asia exhibited rising ASRs. Decomposition analysis indicated that population growth and aging were the main contributors to increased disease burden across different SDI regions. In more developed regions, epidemiological trends had a negative impact on ASMR and age-standardized DALYs rate, whereas in less developed regions, they had a positive impact. Frontier analysis highlighted increasing disparities in disease burden between countries as SDI levels rose, with higher SDI regions having greater potential for reducing disease burden. Projections suggest that ASIR will stabilize by 2040, while ASMR and age-standardized DALYs rate will continue to decline significantly.

Studies have shown that in 2021, the ASIR of CRC exhibited significant regional differences globally. Regions with higher ASIR were concentrated in Europe, North America, Oceania, and developed countries and territories in East Asia such as Japan and South Korea. In contrast, regions with lower ASIR were mainly located in economically underdeveloped areas such as South Asia and Africa. This distribution pattern aligns with previous research findings (30–32). The high ASIR in regions with a high SDI is closely associated with unhealthy lifestyles, dietary habits, and population aging (33). The typical Western dietary pattern, characterized by high caloric, high fat, and high protein intake with insufficient consumption of fiber, grains, fruits, and vegetables, is a significant risk factor for colorectal cancer (34). Additionally, smoking, alcohol consumption, obesity, and lack of physical activity have also been confirmed to increase the risk of CRC, and these adverse factors are particularly prevalent in developed regions (35–37). Decomposition analysis in this study also revealed that population aging has driven the increase in new CRC cases, with aging being a particularly prominent issue in developed regions (38). Moreover, these regions tend to have more advanced screening and reporting systems, resulting in lower rates of missed diagnoses and relatively higher diagnostic rates.

Although developed regions are high-incidence areas for CRC, the improvement in disease burden has been more significant compared to less-developed regions. For example, countries like the United States, Australia, and European nations have effectively reduced the ASIR of CRC through health promotion measures such as smoking and alcohol control, increased dietary fiber intake, encouraging physical exercise, and managing metabolic-related diseases (32). Additionally, the widespread implementation of CRC screening and early-stage surgical interventions in these countries has had a profound impact on long-term disease control (32). However, in recent years, the incidence of CRC has been rising steadily in less-developed regions such as Africa, East Asia, and Latin America. With industrialization and economic development, the typical Western dietary pattern, characterized by high fat, high red meat consumption, and low fiber intake, has become increasingly prevalent, contributing significantly to the rising incidence (30, 36, 39). As the world’s largest developing country, China accounts for approximately 30% of global CRC cases, ranking first worldwide. This is primarily due to the intensifying Westernization of lifestyles, accelerated population aging, and the impacts of industrialization (40). Against the backdrop of population growth and aging, the number of CRC patients in China is expected to increase further in the future. However, China’s CRC screening efforts are still in their early stages, with low coverage and participation rates (41). Therefore, China urgently needs to optimize screening policies, promote non-invasive screening methods, enhance public acceptance, and expand screening coverage to effectively address the challenges posed by CRC incidence.

In this study, regions with a high SDI experienced the most significant reductions in ASMR and age-standardized DALYs rate for CRC, particularly in Australasia, Western Europe, and high-income North America. This trend is largely attributed to the widespread implementation of screening policies in these areas. In most high-SDI countries, regular CRC screening is typically recommended for individuals aged 50 to 75, enabling early diagnosis and treatment, which is one of the key factors behind the notable decline in ASMR (42). For example, since 1998, the United States has actively promoted CRC screening, establishing dedicated multidisciplinary task forces (32). Since the 2000s, colonoscopy has been widely adopted for population screening (43). In Australia, the initiation and continual refinement of the National Bowel Cancer Screening Program since 2006 have significantly reduced mortality rates from CRC (44). In Belgium, Western Europe, the promotion of fecal occult blood testing since 2009 and the expansion of fecal immunochemical testing coverage in 2016 have effectively increased early diagnosis rates (45). These early screening policies enable patients to receive standardized and comprehensive treatment at an early stage of the disease. Furthermore, recent advancements in CRC treatments, including improved surgical techniques, radiotherapy, chemotherapy, targeted therapy, and palliative care, have significantly extended patients’ survival periods (46–49). Compared to less developed regions, patients in high-SDI countries benefit more substantially from the dividends of technological progress, which is a critical factor in the rapid decline of ASMR and age-standardized DALYs rates in these regions. However, despite these significant decreases, the burden of CRC in these regions remains relatively high on a global scale, warranting continued efforts in the future.

In stark contrast to the declining trend in high-SDI regions, ASMR and age-standardized DALYs rates for CRC are rapidly increasing in Southeast Asia, Latin America, and Africa. This growth can be attributed to two primary factors. First, influenced by Western dietary habits and lifestyles, these regions are experiencing a continuous rise in CRC incidence, while the lack of robust screening systems results in low early diagnosis rates (32). Additionally, the relatively underdeveloped healthcare systems in these regions make it difficult for patients to access quality medical resources. Second, economic development has led to improvements in cancer reporting systems, higher patient consultation rates, and reduced underreporting, which have also contributed to the observed data growth. To reduce ASMR and age-standardized DALYs rates in these regions, improving healthcare standards and promoting early screening are undoubtedly critical. However, given the lower economic development levels, achieving these goals independently is challenging. Therefore, we call on capable nations to provide greater medical assistance to low-income countries to collectively alleviate the global burden of CRC. At the same time, these regions can adopt cost-effective measures, such as raising public health awareness through health education, promoting healthy dietary habits to reduce risk factors, and implementing low-cost screening methods like fecal occult blood tests in high-incidence areas to increase early diagnosis rates. These measures will play a vital role in reducing the global burden of CRC.

Studies have shown significant gender differences in the global burden of CRC. Males exhibit higher ASIR, ASMR, and age-standardized DALYs rate compared to females. In 2021, the ASIR for males was 1.58 times that of females. This disparity is closely linked to higher visceral fat prevalence, smoking rates, and alcohol consumption among males (50–52). Additionally, research indicates that endogenous estrogen and oral contraceptives have a protective effect for females, reducing CRC incidence, which partially explains the ASIR gap between genders (53, 54). Furthermore, this study found that the improvement in ASIR among females significantly outpaced that of males, indicating that the ASIR gap may continue to widen in the future, a trend that deserves close attention. Beyond ASIR differences, females also exhibit significantly lower ASMR and age-standardized DALYs rates compared to males. Some studies suggest that gender differences in gut microbiota and metabolites may be key factors (55). Male-biased gut metabolites exacerbate colorectal tumor development through the glycerophospholipid metabolic pathway, potentially contributing to higher CRC mortality in males.

The study, through decomposition analysis, explored the roles of population growth, population aging, and changes in epidemiological trends on the variations in new CRC cases, deaths, and DALYs from 1990 to 2021. The results showed that population growth and aging were the main drivers of increases in new cases, deaths, and DALYs. As age advances, the risk of chronic diseases such as hyperlipidemia and hypertension, which are known risk factors for CRC, increases (56). This highlights the crucial role of population aging. Furthermore, changes in epidemiological trends in high SDI, medium SDI, and low-middle SDI groups contributed to the rise in new cases. This is likely due to accelerated industrialization, rapid economic transitions, and the growing prevalence of CRC risk factors in these regions (30). In contrast, in high-SDI areas, epidemiological trends negatively impacted new case numbers, primarily due to early screening and timely removal of precancerous lesions. Meanwhile, in low-SDI regions, the negative effect of epidemiological trends on new cases might stem from lower levels of economic development, slower industrialization, and reduced exposure to risk factors. However, this does not mean that reducing economic development would lower the CRC burden. Frontier analysis suggested that underdeveloped regions achieved lower optimal ASIR levels, which is likely related to outdated medical technologies and health systems. The study also found that as economic development improved, changes in epidemiological trends had a more pronounced effect in reducing CRC deaths and DALYs. This improvement was attributed to advancements in screening systems and treatment methods, further supporting the findings of the frontier analysis that developed countries are capable of achieving lower ASMR and DALY rates.

The study also used the BAPC model to predict trends in CRC ASRs through 2040. It found that the ASMR and age-standardized DALYs rates for CRC are expected to decline across all genders, as well as for males and females individually. Although this indicates progress in disease control efforts, it is primarily attributed to advancements in developed regions, while the contributions from underdeveloped regions remain limited. This underscores the need for focused prevention and control efforts in underdeveloped areas in the future. Regarding ASIR, the study revealed that although the ASIR for females is expected to decline, the ASIR for males and the overall population is projected to increase. This trend is strongly linked to higher smoking and alcohol consumption rates among males. These findings highlight the importance of targeted prevention strategies for the male population, including health education initiatives aimed at reducing exposure to colorectal cancer risk factors.

Our study indicates that by 2040, the global ASIR for CRC will stabilize, while the ASMR and age-standardized DALYs rate will show a declining trend. While progress has been made in CRC control, significant challenges remain. In developed regions, the disease burden remains heavy, and the increasing population and aging issues may further increase incident cases, mortality, and DALYs in the future, posing a major challenge. For less developed regions, the lower disease burden might be due to low diagnostic rates from poorer medical standards. Furthermore, detection and screening are inadequate, and CRC is often diagnosed at advanced stages with poorer local medical conditions, leading to higher mortality and shorter life expectancy. As unhealthy dietary habits spread in less developed regions, such as lo7w intake of fruits and vegetables, prolonged sedentary behavior, and lack of exercise, improving screening systems and medical standards to increase early diagnosis rates, prolong survival, and promote prevention campaigns is crucial (30). Thus, the rising burden of CRC in areas with low SDI requires targeted interventions. We recommend prioritizing the integration of CRC screening into public health services, reducing costs through government subsidies and insurance, especially by setting up mobile screening vans in remote communities, and introducing low-cost screening tools (e.g., fecal occult blood testing) to reduce the proportion of late confirmed diagnoses. At the same time, localized health education is promoted, working with community leaders to publicize CRC risk factors and spreading health literacy in schools. In terms of medical facilities, additional CRC diagnostic equipment should be installed, primary healthcare workers should be trained to identify high-risk groups, and the role of “CRC health coach” should be established to be responsible for follow-up visits and regular screening. It is recommended that a CRC data monitoring system be established to help policymakers grasp the dynamics of the burden and intervene in a timely manner. Through these measures, low-SDI regions can more effectively control the burden of CRC and provide better health protection for their residents. In addition, in our frontier analysis, we found that regions with high SDI could achieve lower levels of CRC burden, which we believe may be related to better cancer screening systems with high SDI and greater access to medical resources for patients. Therefore, efforts to improve their own economic development level is also an important way to reduce the burden of CRC.

Using the latest data from GBD 2021, our study provides a comprehensive evaluation of the global burden and trends of CRC from 1990 to 2021. However, our study has certain limitations. First, CRC exhibits significant differences in anatomical locations and genetic characteristics, which influence its clinical manifestations, treatment, and prognosis (57). It can be subdivided into right-sided colon cancer, left-sided colon cancer, and rectal cancer. Right-sided colon cancer typically occurs in the cecum and ascending colon, where the larger lumen often leads to subtle early symptoms, commonly presenting as anemia and fatigue, with a relatively poorer prognosis. In contrast, left-sided colon cancer, encompassing the descending colon and sigmoid colon, often manifests earlier with symptoms such as hematochezia and bowel obstruction. Rectal cancer, due to its proximity to the anus, frequently requires a comprehensive treatment approach involving surgery, chemotherapy, and radiation therapy. The genetic differences in CRC are reflected in its molecular subtypes, such as chromosomal instability (CIN) and microsatellite instability (MSI) (58). CIN-type cancers are characterized by numerous chromosomal rearrangements and mutations in key genes like APC, KRAS, and TP53, and tend to be more aggressive, whereas MSI-type cancers exhibit a high mutation burden associated with defects in DNA mismatch repair, responding favorably to immune checkpoint inhibitors (58, 59). Secondly, the BAPC model used for predictions relies solely on demographic variables (age, period, cohort) and does not account for changes in modifiable risk factors, such as dietary patterns, smoking rates, or access to healthcare. This narrow approach may lead to an underestimation of future CRC burden, particularly in low- and middle-income countries where rapid economic development is driving the adoption of Westernized lifestyles. Third, the accuracy and robustness of GBD-based studies are heavily reliant on the quality of primary epidemiological data collection. However, in certain countries and regions, limitations in data availability can undermine the reliability and precision of the modeling. Specifically, many low- and middle-income countries lack comprehensive population-based cancer registries, or in some cases, any registry system at all. This data gap poses significant challenges to producing accurate and representative estimates. Finally, although this study explored the effect of SDI on the burden of CRC, SDI may not fully reflect other important factors that influence mortality or DALYs rates, such as access to healthcare, screening, and treatment. Therefore, the ability of future studies to provide more relevant data will facilitate in-depth analysis and more accurate assessment of the role of these factors.

In the context of GBD database research on CRC, several promising avenues deserve exploration. First, future research should aim to improve the data quality of the global disease burden database, particularly focusing on areas with missing or incomplete data. Enhancing international cooperation and standardizing data collection methods can improve data accuracy and consistency. Second, integrating multi-omics data, including genomics, transcriptomics, pathological types, and proteomics, into the GBD framework can promote understanding of CRC pathogenesis. Third, artificial intelligence and machine learning technologies have broad prospects in medical data analysis. These technologies could play crucial roles in CRC risk prediction, disease screening, and personalized treatment plan design, advancing CRC prevention and treatment into an intelligent era. Lastly, developing and using more flexible dynamic data updating and real-time analysis tools can more rapidly capture and reflect global CRC burden changes, facilitating timely adjustments in public health strategies and interventions.

## 5 Conclusion

With the increasing incident cases, deaths, and DALYs of CRC globally, it has become a significant cause of cancer-related deaths and disease burden worldwide. Globally, the disease burden of CRC mainly comes from developed countries, and it is expected to increase furthe. However, ASRs in developed countries have begun to decline, extending the life expectancy of CRC patients. In less developed regions, such as Africa, South Asia, and Southeast Asia have a significant increase in ASIR. Considering the economic situation of low developed countries and regions, it is impossible to achieve a wide range of disease screening, we suggest that some low-cost screening methods such as fecal blood examination can be carried out in areas with a high incidence of CRC, and further examination can be conducted for highly skeptical people. At the same time, targeted education work can also be carried out according to the risk factors of local CRC to raise people’s awareness of prevention. More rigorous methods for studying the effectiveness of these interventions in low-resource settings are highly anticipated in the future, as well as improved data collection to better understand local risk factors and health status in these areas.

## Data Availability

The original contributions presented in the study are included in the article/**Supplementary Material**. Further inquiries can be directed to the corresponding author.
