# Supplementary material for: Global, regional, and national trends in colorectal cancer burden from 1990 to 2021 and projections to 2040
Source: Front Oncol. 2025 Jan 16;14:1466159. doi: 10.3389/fonc.2024.1466159 (PMC11779618; doi:10.3389/fonc.2024.1466159)
Supplement: Supplementary file 1 [file Table1.docx]

**Supplementary Methods**

1. **Decomposition analysis**

This study uses decomposition analysis based on the Das Gupta method to illustrate the roles of epidemiological changes, population growth, and population aging in driving changes in new cases, deaths, and DALYs from 1990 to 2021(1, 2). We then present the analysis process using global new case numbers as an example, designed with reference to previous literature(3).

a_effect <- (sum(a_2021*p_1990*r_1990) + sum(a_2021*p_2021*r_2021))/3 +

(sum(a_2021*p_1990*r_2021) + sum(a_2021*p_2021*r_1990))/6 -

(sum(a_1990*p_1990*r_1990) + sum(a_1990*p_2021*r_2021))/3 -

(sum(a_1990*p_1990*r_2021) + sum(a_1990*p_2021*r_1990))/6

p_effect <- (sum(a_1990*p_2021*r_1990) + sum(a_2021*p_2021*r_2021))/3 +

(sum(a_1990*p_2021*r_2021) + sum(a_2021*p_2021*r_1990))/6 -

(sum(a_1990*p_1990*r_1990) + sum(a_2021*p_1990*r_2021))/3 -

(sum(a_1990*p_1990*r_2021) + sum(a_2021*p_1990*r_1990))/6

r_effect <- (sum(a_1990*p_1990*r_2021) + sum(a_2021*p_2021*r_2021))/3 +

(sum(a_1990*p_2021*r_2021) + sum(a_2021*p_1990*r_2021))/6 -

(sum(a_1990*p_1990*r_1990) + sum(a_2021*p_2021*r_1990))/3 -

(sum(a_1990*p_2021*r_1990) + sum(a_2021*p_1990*r_1990))/6

The code above demonstrates how we use R software to calculate the effects of population aging, population growth, and epidemiological trend changes on the global changes in new colorectal cancer cases from 1990 to 2021. Here, a_effect, p_effect, and r_effect represent the effects of population aging, population growth, and epidemiological trends, respectively, on the change in new cases. In our study, we divided the population into 20 age groups, from <5 years to >95 years, in 5-year increments. a_1990 and r_1990 are vectors of 20 elements, representing the colorectal cancer incidence rate for each age group globally and the proportion of each age group within the total population in 1990, respectively, while p_1990 denotes the total global population in 1990. Similarly, a_2021, p_2021, and r_2021 represent the same parameters for 2021. By dividing a_effect, p_effect, and r_effect by the total effect (the sum of these three), we can determine the proportional contribution of each factor to the overall effect.

1. **Frontier analysis**

To assess the relationship between colorectal cancer burden and sociodemographic development, we applied frontier analysis as a quantitative method, using Socio-demographic Index (SDI) as a measure of development level to establish the minimum achievable ASIR, ASMR, and age-standardized DALYs rate(4). Next, we illustrate this using ASIR as an example. The ASIR frontier defines the lowest ASIR that a country or region could achieve at a given SDI level. The distance to the frontier, known as the efficiency gap, indicates potential unrealized gains or improvement opportunities. The greater the distance from the frontier, the higher the potential for benefit at that SDI level. To depict the nonlinear frontier, we utilized Data Envelopment Analysis (DEA) with the Free Disposal Hull (FDH) method to construct an SDI-adjusted frontier for colorectal cancer ASIR(3). This analysis incorporated data spanning from 1990 to 2021. To account for uncertainty, we performed random resampling of data across all countries and years to generate 1,000 bootstrap samples. We calculated the average ASIR of colorectal cancer for each SDI value, and then applied LOESS regression with a degree of 1 and a span of 0.2 to produce a smooth frontier line.

1. **Age-Period-Cohort Model**

The Bayesian Age-Period-Cohort Model (BAPC) is a statistical model used to analyze and predict the influence of age, time period, and birth cohort on specific events, such as mortality or disease incidence, within population data(5, 6). This model leverages the advantages of Bayesian statistical methods, allowing it to handle complex data structures and account for uncertainty. The following analysis provides a detailed demonstration of the process, using the prediction of global, all-gender colorectal cancer ASIR as an example.

To begin, a data frame named data was created, with 51 rows and 20 columns. The rows represent the years from 1990 to 2040, while the columns denote 20 age groups ranging from "<5 years" to "95+ years," with each column representing a 5-year interval. This table contains data on the number of new colorectal cancer cases among all age groups globally, from 1990 to 2040. Next, a second data frame, population, was created with the same dimensions (51 rows and 20 columns) and row/column names as data. This table represents the global population count within each age group. Subsequently, the global population proportion for each age group (from "<5 years" to "95+ years") was obtained, showing the percentage of the global population within each age group. The projected population data used in this study is provided by the Institute for Health Metrics and Evaluation (IHME) and can be accessed at <https://ghdx.healthdata.org/download-access/login>. Finally, the BAPC and INLA packages in R were used to fit the model to the data(7).

**References**

1. Das Gupta P. Standardization and decomposition of rates from cross-classified data. Genus. 1994;50(3-4):171-96.

2. Chevan A, Sutherland M. Revisiting Das Gupta: refinement and extension of standardization and decomposition. Demography. 2009;46(3):429-49.

3. Xie Y, Bowe B, Mokdad AH, Xian H, Yan Y, Li T, et al. Analysis of the Global Burden of Disease study highlights the global, regional, and national trends of chronic kidney disease epidemiology from 1990 to 2016. Kidney Int. 2018;94(3):567-81.

4. Sultana S, Hossain MM, Haque MN. Estimating the potato farming efficiency: A comparative study between stochastic frontier analysis and data envelopment analysis. PLoS One. 2023;18(4):e0284391.

5. Knorr-Held L, Rainer E. Projections of lung cancer mortality in West Germany: a case study in Bayesian prediction. Biostatistics. 2001;2(1):109-29.

6. Berzuini C, Clayton D. Bayesian analysis of survival on multiple time scales. Stat Med. 1994;13(8):823-38.

7. Knoll M, Furkel J, Debus J, Abdollahi A, Karch A, Stock C. An R package for an integrated evaluation of statistical approaches to cancer incidence projection. BMC Med Res Methodol. 2020;20(1):257.
