# Supplementary material for: Global, regional, and national trends in colorectal cancer burden from 1990 to 2021 and projections to 2040
Source: Front Oncol. 2025 Jan 16;14:1466159. doi: 10.3389/fonc.2024.1466159 (PMC11779618; doi:10.3389/fonc.2024.1466159)
Supplement: Supplementary file 4 [file DataSheet1.docx]

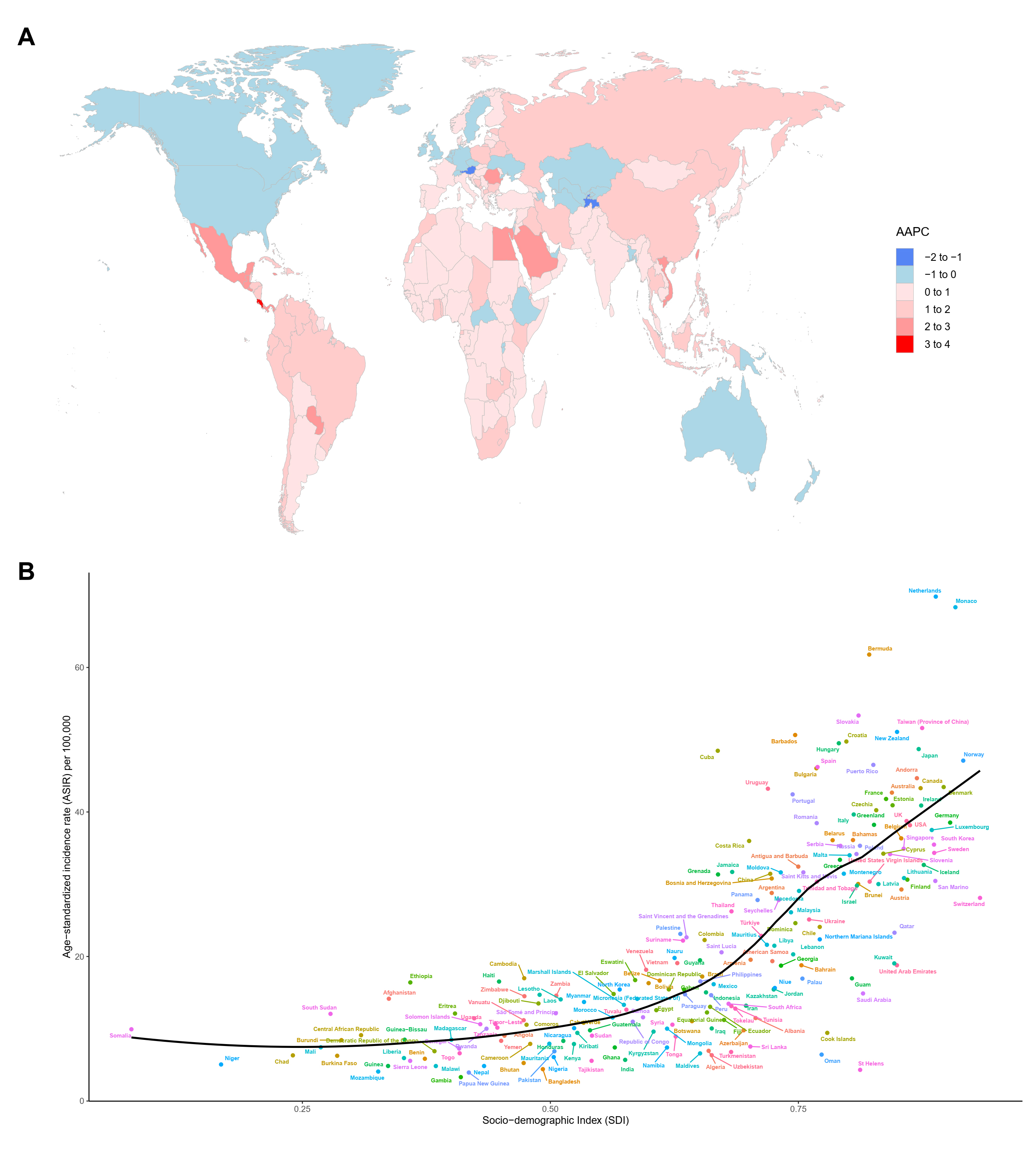


Supplementary Figure 1. Multidimensional analysis of ASIR of CRC. A. AAPC of ASIR from 1990 to 2021 across 204 countries and territories; B: Relationship between the SDI and ASIR in 204 countries and territories in 2021. AAPC, average annual percentage change; ASIR, age-standardized incidence rate; SDI, Socio-demographic index; CRC, colorectal cancer.


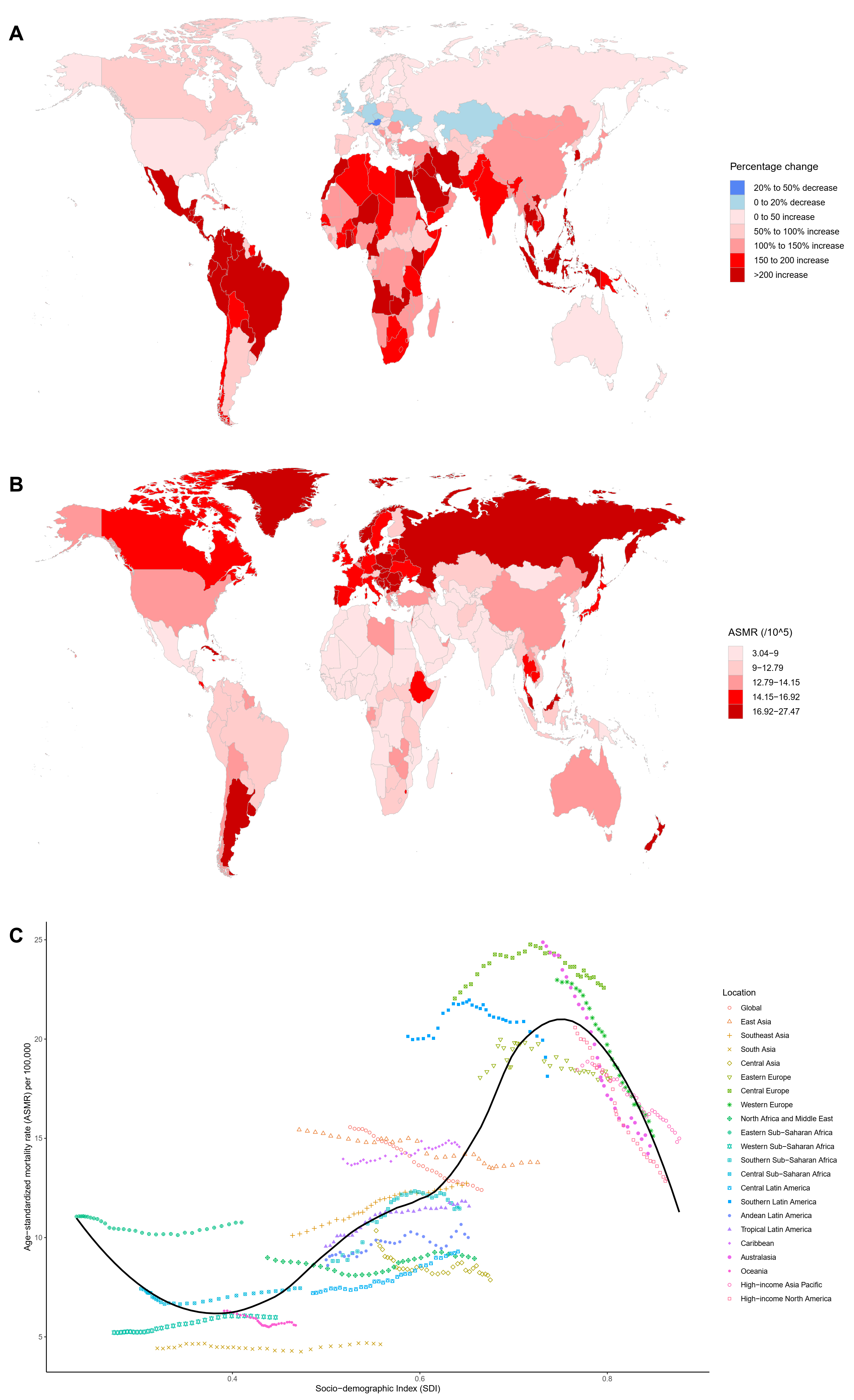


Supplementary Figure 2: Multidimensional analysis of CRC mortality. A. Percentage change in the number of death cases from 1990 to 2021 across 204 countries and territories; B. ASMR in 204 countries and territories in 2021; C. Association between the SDI and ASMR in 21 GBD regions from 1990 to 2021. ASMR, age-standardized mortality rate; SDI, Socio-demographic index; CRC, colorectal cancer; GBD, Global Burden of Diseases, Injuries, and Risk Factors.


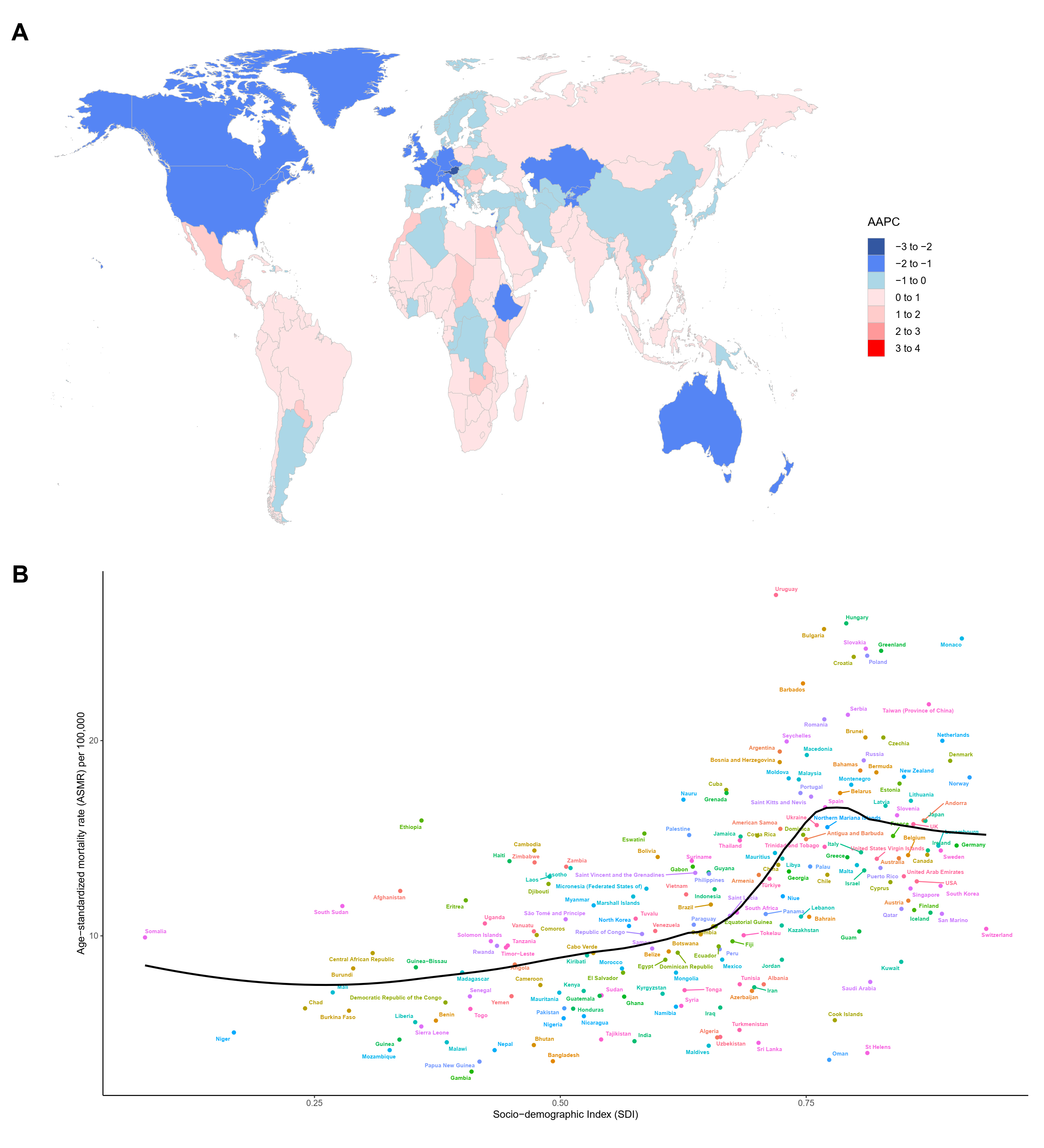


Supplementary Figure 3. Multidimensional analysis of ASMR of CRC. A. AAPC of ASMR from 1990 to 2021 across 204 countries and territories; B: Relationship between the SDI and ASMR in 204 countries and territories in 2021. AAPC, average annual percentage change; ASMR, age-standardized mortality rate; SDI, Socio-demographic index; CRC, colorectal cancer.


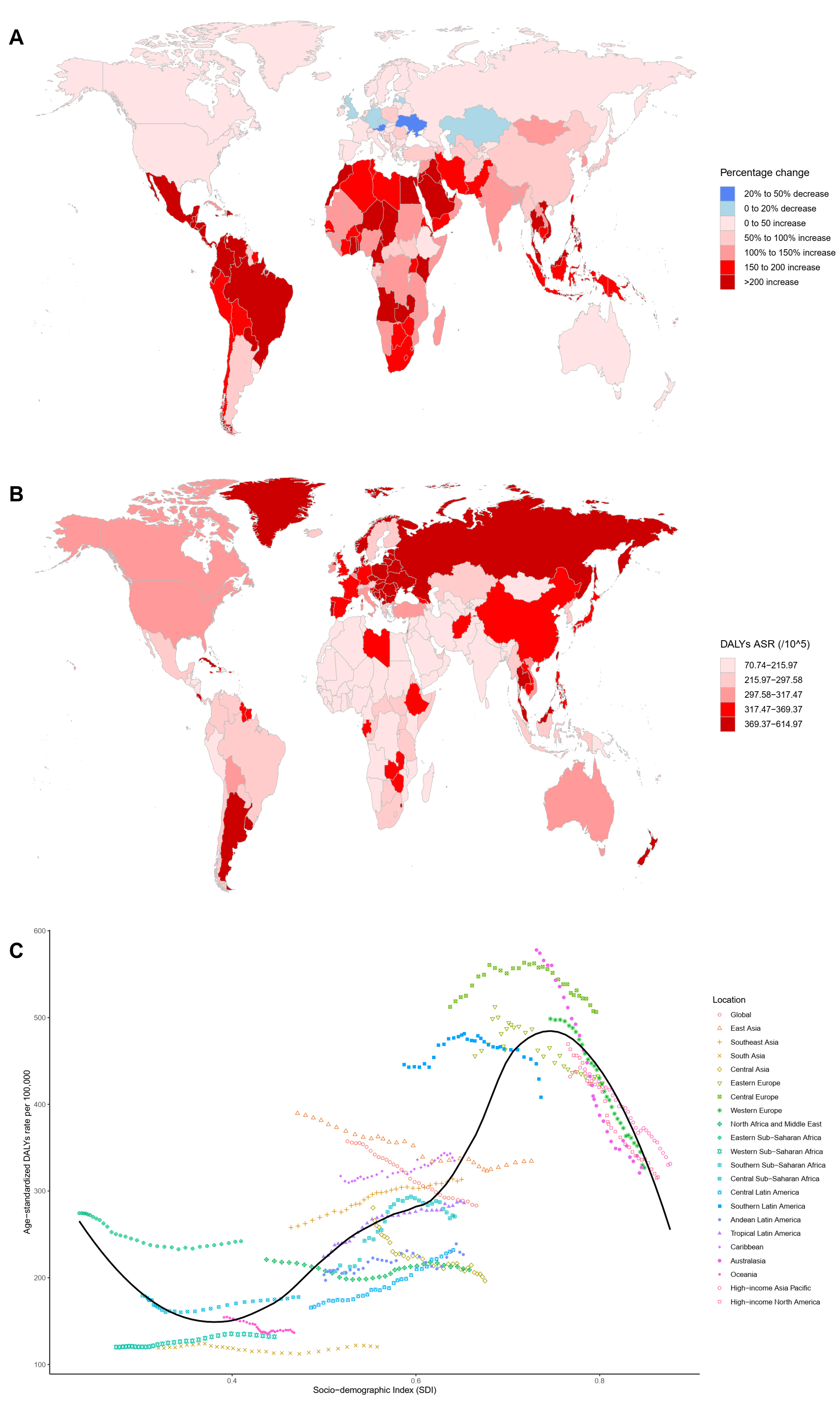


Supplementary Figure 4: Multidimensional analysis of CRC DALYs. A. Percentage change in the number of DALYs from 1990 to 2021 across 204 countries and territories.; B. age-standardized DALYs rate in 204 countries and territories in 2021; C. Association between the SDI and age-standardized DALYs rate in 21 GBD regions from 1990 to 2021. DALYs, disability-adjusted life years; ASR, age-standardized rate; SDI, Socio-demographic index.


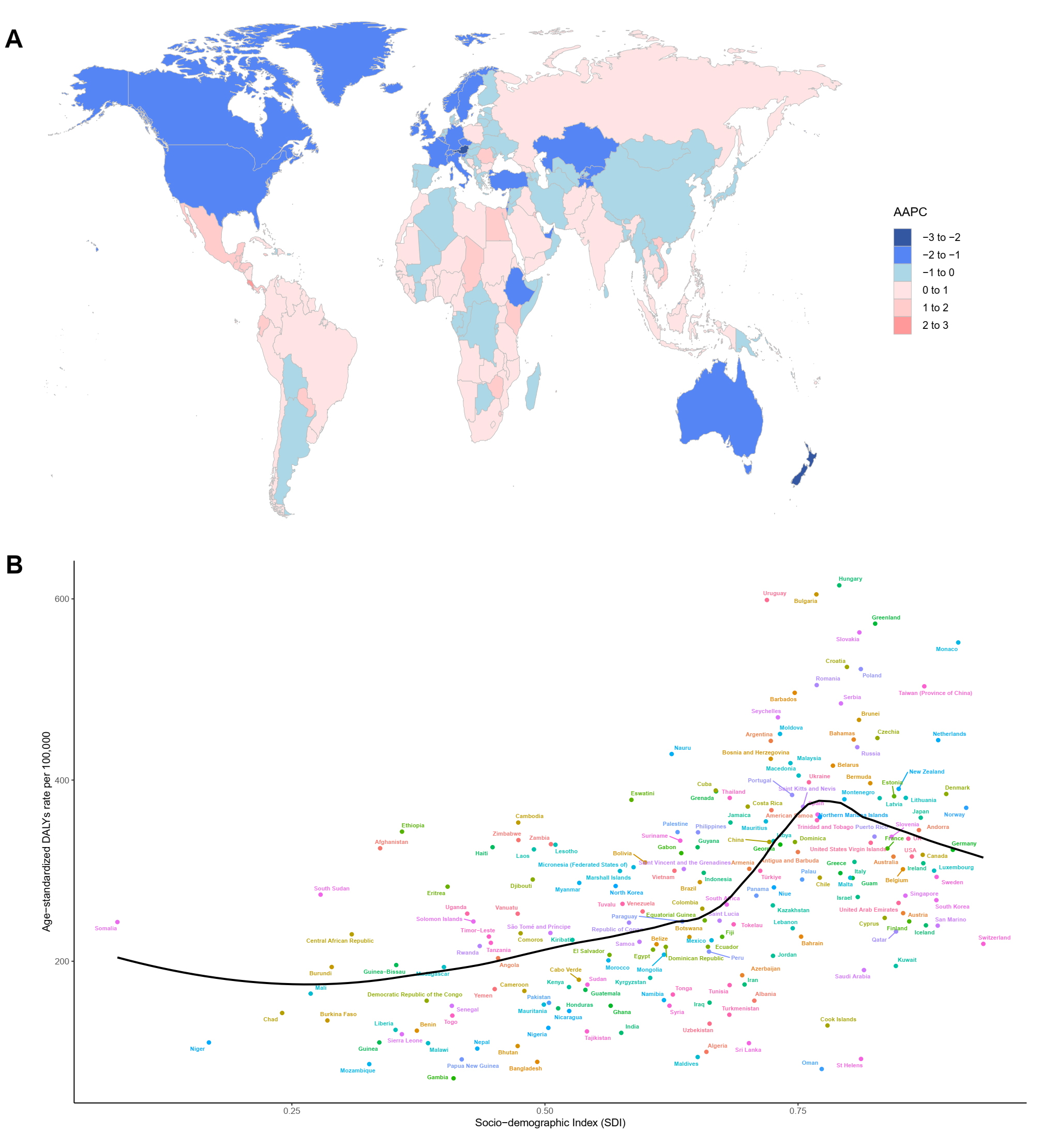


Supplementary Figure 5. Multidimensional analysis of age-standardized DALYs rate of CRC. A. AAPC of age-standardized DALYs rate from 1990 to 2021 across 204 countries and territories; B. Relationship between the SDI and age-standardized DALYs rate in 204 countries and territories in 2021. AAPC, average annual percentage change; DALYs, disability-adjusted life years; SDI, Socio-demographic index; CRC, colorectal cancer.


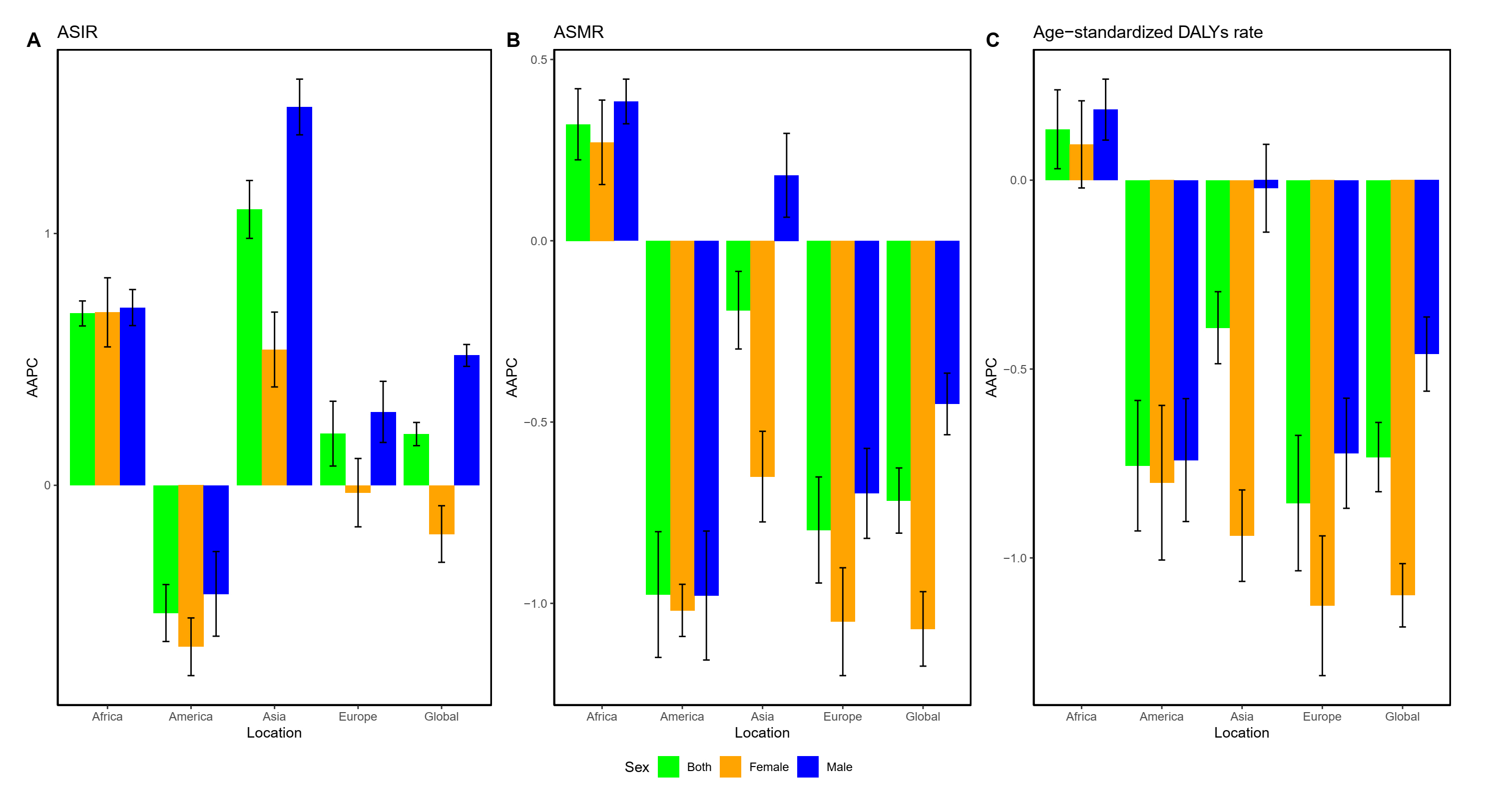


Supplementary Figure 6: AAPC of CRC by gender and continents from 1990 to 2021. A. Analysis of ASIR; B. Analysis of ASMR; C. Analysis of age-standardized DALYs rate. ASIR, age-standardized incidence rate; ASMR, age-standardized mortality rate; DALYs, disability-adjusted life years; AAPC, average annual percentage change; CRC, colorectal cancer.


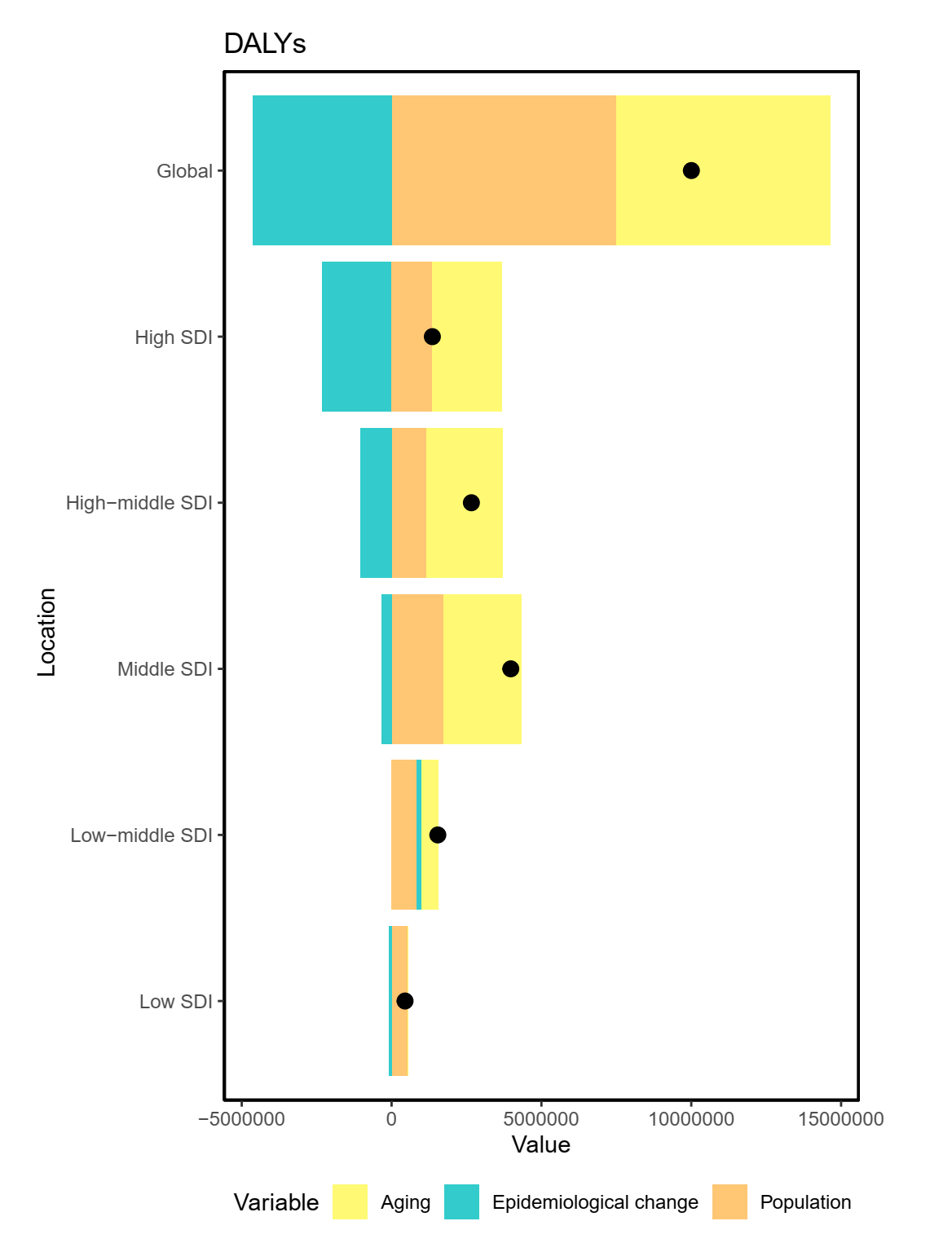


Supplementary Figure 7. Decomposition analysis of global CRC DALYs from 1990 to 2021. Black dots represent the overall changes in disease burden due to aging, epidemiological changes, and population growth. For each component, an increase in the disease burden of CRC related to that component is indicated by positive values, whereas a decrease is indicated by negative values. DALYs, disability-adjusted life years; SDI, socio-demographic index; CRC, colorectal cancer.


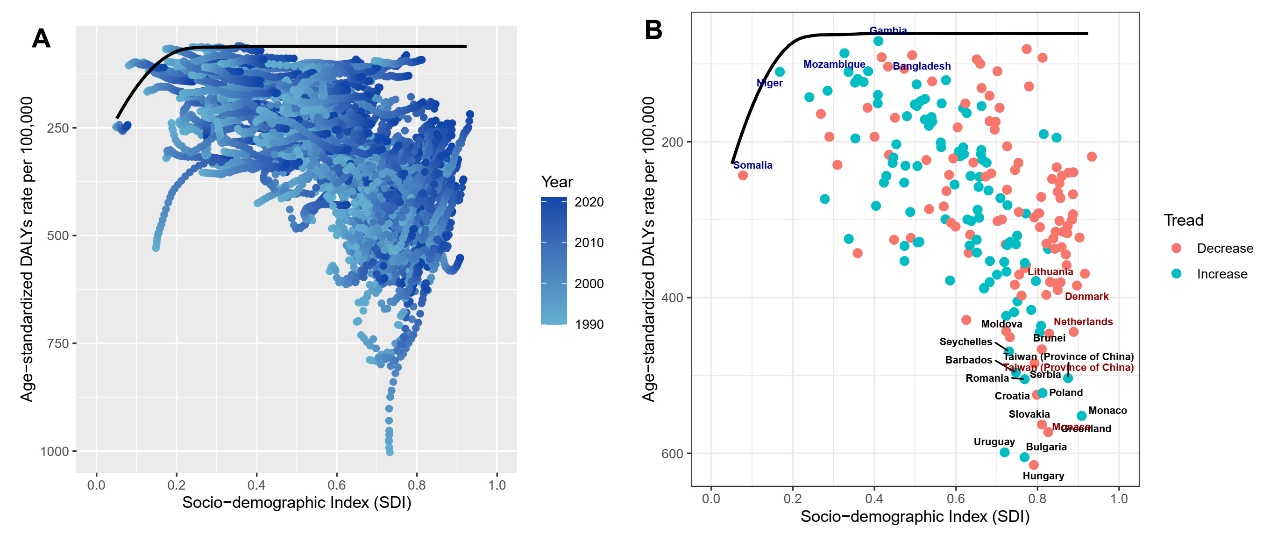


Supplementary Figure 8. Frontier analysis of global CRC DALYs from 1990 to 2021. Black lines represent the lower limits of ASR achievable at different SDI levels, with points representing different countries and regions. In Figures A, blue dots are used, with the color deepening to indicate the progression of years. In panels B, blue dots indicate countries and territories with increased ASRs from 1990 to 2021, while red dots indicate those with decreased ASRs. The 15 countries and regions with the largest effective differences globally are labeled in black font, the 5 countries and regions with the smallest effective differences among low SDI countries are labeled in blue font, and the 5 countries and regions with the largest effective differences among high SDI countries are labeled in red font. DALYs, disability-adjusted life years; SDI, Socio-demographic index; CRC, colorectal cancer; ASR, age-standardized rate.
